# A comparison of RNA amplification techniques at sub-nanogram input concentration

**DOI:** 10.1186/1471-2164-10-326

**Published:** 2009-07-20

**Authors:** Julie E Lang, Mark Jesus M Magbanua, Janet H Scott, G Mike Makrigiorgos, Gang Wang, Scot Federman, Laura J Esserman, John W Park, Christopher M Haqq

**Affiliations:** 1 Department of Surgery, UCSF Comprehensive Cancer Center, 1500 Divisadero Street, San Francisco, CA 94143, USA; 2 Department of Medical Oncology, UCSF Comprehensive Cancer Center, San Francisco, CA 94143, USA; 3 Department of Urology, UCSF Comprehensive Cancer Center, San Francisco, CA 94143, USA; 4 Dana Farber Cancer Institute, Harvard Medical School, 44 Binney Street, Boston, MA 02115, USA; 5 Department of Surgery, Arizona Cancer Center, University of Arizona, 1515 N, Campbell Ave #1968, PO Box 245024, Tucson, AZ 85724, USA

## Abstract

**Background:**

Gene expression profiling of small numbers of cells requires high-fidelity amplification of sub-nanogram amounts of RNA. Several methods for RNA amplification are available; however, there has been little consideration of the accuracy of these methods when working with very low-input quantities of RNA as is often required with rare clinical samples. Starting with 250 picograms-3.3 nanograms of total RNA, we compared two linear amplification methods 1) modified T7 and 2) Arcturus RiboAmp HS and a logarithmic amplification, 3) Balanced PCR. Microarray data from each amplification method were validated against quantitative real-time PCR (QPCR) for 37 genes.

**Results:**

For high intensity spots, mean Pearson correlations were quite acceptable for both total RNA and low-input quantities amplified with each of the 3 methods. Microarray filtering and data processing has an important effect on the correlation coefficient results generated by each method. Arrays derived from total RNA had higher Pearson's correlations than did arrays derived from amplified RNA when considering the entire unprocessed dataset, however, when considering a gene set of high signal intensity, the amplified arrays had superior correlation coefficients than did the total RNA arrays.

**Conclusion:**

Gene expression arrays can be obtained with sub-nanogram input of total RNA. High intensity spots showed better correlation on array-array analysis than did unfiltered data, however, QPCR validated the accuracy of gene expression array profiling from low-input quantities of RNA with all 3 amplification techniques. RNA amplification and expression analysis at the sub-nanogram input level is both feasible and accurate if data processing is used to focus attention to high intensity genes for microarrays or if QPCR is used as a gold standard for validation.

## Background

Expression array analysis has provided valuable new insights into the biology and pathophysiology of many cancers [1-4]. However, initial studies required relatively large amounts of tumor tissue (typically 4–40 ug of total RNA). It is important to extend these powerful analytical methods to much smaller quantities of cells, such as rare clinical specimens, including small tumors, core biopsies, or even individual cells. Working with limited amounts of RNA does introduce problems related to signal versus noise amplification[5]. This is relevant when considering expression profiling of samples from needle biopsies, rare tissue samples and pure cellular preparations and planning experimental design and data analysis.

Feasibility and reproducibility has been established for linear amplification from nanogram to low microgram input quantities of total RNA to yield high fidelity gene amplification products suitable for gene expression microarray analysis [6-16]. In an experimental model using reverse transcribed product diluted down to the sub-nanogram range, Subkhankulova et al were the first to report comparisons of amplification techniques at the sub-nanogram level[17]. They compared 1) T7 based linear amplification to 2) switching mechanism at 5'end of RNA template (SMART) PCR and 3) global PCR amplification[17]. Surprisingly, they found that PCR amplification was more reliable than linear amplification for detecting true expression differences between picogram input samples with higher correlation between technical replicates than linear amplification. Unfortunately, this higher true-positive rate was at the expense of a considerably decreased absolute discovery rate. In contrast, Wang et al compared T7 based linear amplification to SMART PCR and found that while both methods achieved reproducible, reliable results that the T7 based method yielded more amplified RNA and is therefore preferred when the amount of starting total RNA is limited[18] We evaluated the hypothesis that high fidelity amplification is possible by both linear and PCR based methods when starting with samples containing sub-nanogram amounts of total RNA.

## Methods

To test our hypothesis, serial dilutions of stock RNA was employed for a comparison of 3 amplification techniques with assessment by technical replicates of microarrays and with subsequent validation by QPCR of both total and amplified RNA.

### RNA Isolation

Total RNA was prepared from the BT474 cell line and Stratagene Universal Human Reference RNA (StratRef) (Stratagene, La Jolla, CA) using the Arcturus PicoPure RNA isolation kit (Mountain View, CA) as per manufacturer's instructions. StratRef is composed of total RNA from 10 human cell lines and is designed to be used as a reference for microarray gene-profiling experiments. Serial dilutions of StratRef and BT474 RNA served as the substrate for all amplification reactions to minimize sources of variability.

### Total RNA labeling without amplification

10 μg of total RNA from the BT474 breast cancer cell line and StratRef RNA was reverse transcribed using Stratascript RT (Stratagene, La Jolla, CA) in the presence of 10 μg of random hexamer (Amersham Pharmacia) and oligo d(T)_24_NN (sequence provided in table 1) using the reverse transcriptase manufacturer's recommended protocol.

**Table 1:** Lower limits of total RNA required for each amplification method.

**Amplification technique Modified T7**	**Amplification yield, BT474 RNA(mcg)**
140 pg	0.80 – insufficient
280 pg	0.60 – insufficient
375 pg	0.16 – insufficient
500 pg	1.4 – sufficient for 1 array
500 pg	1.7 – sufficient for 1 array
500 pg	0 – insufficient
500 pg	0 – insufficient
500 pg	1.9 – sufficient for 1 array
1 ng	17.8
1 ng	20.3
**Arcturus**	
100 pg	0 – insufficient
100 pg	0 – insufficient
250 pg	12
250 pg	7.2
500 pg	56.8
1 ng	52.7
Balanced PCR*	
500 pg	0 – insufficient
500 pg	1.5 – sufficient for 1 array
667 pg	2.1 – sufficient for 1 array
667 pg	2.0 – sufficient for 1 array
1 ng	0 – insufficient
1 ng	2.0 – sufficient for 1 array
3.3 ng	2.3 – sufficient for 1 array
3.3 ng	3.2

*1 array discarded due to poor hybridization

### Modified T7 RNA amplification

Total RNA from the BT474 breast cancer cell line and StratRef was linearly amplified through two rounds of *in vitro *transcription (IVT) based on a modified T7 amplification[19]. Reverse transcription (RT) of total RNA was performed using 100 ng of oligo dT-T7 primer (sequence provided in table 1) First strand synthesis was as described previously[8], however, T4gp32 was added in the first strand synthesis to improve full-length DNA synthesis[20]. Second strand synthesis was performed followed by DNA purification with Zymo DNA Clean and Concentrator kits (Zymo Research, Orange, CA). Two rounds of *in vitro *transcription were performed[8]. Samples were purified with Qiagen RNeasy mini columns (Qiagen, Valencia CA).

### Arcturus HS amplification

Total RNA from the BT474 breast cancer cell line and StratRef (Stratagene, La Jolla, CA), was linearly amplified through two rounds of *in vitro *transcription according to the manufacturer's published instructions (at the time of these experiments reagents were produced by Arcturus, Mountain View, CA, now MDS Analytical Technologies, Sunnyvale, CA). Their protocol specified that a minimum input of 100–500 picograms of total RNA are required for successful amplification with this kit designed specifically for low-input total RNA samples, which is equivalent to 10–50 cells. Per the manufacturer's specifications, 200 ng of Poly dIdC nucleic acid carrier was added to each reaction. IVT reaction time was 6.5 hours.

### Balanced PCR amplification

Total RNA from the BT474 breast cancer cell line and StratRef (Stratagene, La Jolla, CA), was reverse transcribed using separate oligo dTT7 primers, pooled and exponentially amplified in the same PCR tube[21]. Briefly, the balanced PCR reactions were carried out as follows. We mixed 1 μl of cDNA from the target cells or from the reference with T4 DNA ligase buffer, and restricted it with 0.5 μl of 10 U/μl NlaIII at 37°C for one hour. For digestion with NlaIII, linker LN1 is used for control and LN2 for target cDNA (sequence provided in Table 1). We annealed the appropriate linkers to cDNA by serially decreasing the temperature of the sample from 50°C to 10°C at 5°C ramp in 5 minute steps. We then added 0.5 μl of 2,000 U/μl T4 DNA ligase and incubated at room temperature for 1 hour. We then mixed together the cDNAs ligated to different linkers and purified the mixture with a QIAquick PCR Purification Kit (Qiagen, Valencia CA).

To 20 μl of purified-ligated DNA, we used the Advantage 2 PCR Polymerase system as per manufacturer's instructions (Clontech, Mountain View CA), dNTP mix (10 mM ea.), 1 μl of 10 μM common primer P1 (sequence provided in Table 1) and 22 μl of H2O. PCR was performed at 72°C for 8 minutes; 95°C for 1 minute; 20 cycles of 95°C for 30 seconds and 72°C for 1 minutes; then 72°C for 5 minutes. We purified the PCR product twice with QIAquick PCR Purification Kit (Qiagen, Valencia CA) and eluted the DNA in 50 μl of H2O. We quantified the cDNA concentration with Picogreen (Invitrogen, Carlsbad CA). We mixed 1 μl of 3 ng/μl DNA with 5 μl of 10× TITANIUM™ Taq PCR Buffer (Clontech, Mountain View CA), 1 μl of 50× TITANIUM™ Taq Polymerase (Clontech, Mountain View CA), 1 μl of 50× dNTP Mix (10 mM ea.), 5 μl of 4 μM P2a for LN1-ligated cDNA or P2b for LN2-ligated cDNA, and 37 μl of H2O. We separated and amplified the cDNA at 95°C for 1 minute; 10 cycles of 95°C for 30 seconds and 72°C for 1 minute; and 72°C for 5 minutes. Four reactions were carried out per sample and were pooled.

Although the balanced PCR reactions were carried out in a different laboratory (Makrigiorgos lab, Boston MA) than the linear amplifications (Haqq lab, San Francisco, CA), an aliquot of RNA from the same tube of StratRef RNA and an aliquot of the same preparation of BT474 RNA as was used to minimize input variability. Reverse transcription of this RNA was performed before shipping the cDNA on dry ice for the subsequent Balanced PCR reactions. Coupling, array hybridization and analysis were all performed in the Haqq lab, as were all other techniques described.

### Assessment of Transcript Integrity

The molecular weight profile and integrity of each amplified RNA/DNA species was evaluated using the Agilent Bioanalyzer 2100 (Agilent Technologies, Palo Alto, CA) with a RNA 6000 Pico Lab Chip.

### Fluorescent labeling

1.25 μg of amplified RNAs (aRNAs) produced with Modified T7 and Arcturus RiboAmp HS were converted to amino-allyl modified cDNA and coupled to N-hydroxysuccinimidyl esters of Cy3 or Cy5 (Amersham, Piscataway, NJ)[22]. The Balanced PCR amplified cDNAs (1.25 μg) were labeled with Klenow from BioPrime (Invitrogen, Carlsbad, CA) and Cy3/Cy5 dUTP (Amersham, Piscataway, NJ). All specimens were then hybridized to a microarray slide at 65°C for 12–16 hours. The slide was then washed and immediately scanned with Axon Imager 4000b (Axon Instruments, Union City, CA), utilizing GenePixPro 3.0 software.

### Microarrays

The 20,862 cDNAs used in these studies were from Research Genetics (Huntsville, AL), now Invitrogen (Carlsbad, CA). On the basis of Unigene build 166, these clones represent 19,740 independent loci. Hybridization, washing, scanning and primary data analysis was performed as previously described[23,24]. A total of 17 technical replicate microarrays, including 3 unamplified, 5 T7 amplified, 4 Arcturus amplified and 5 balanced PCR amplified arrays for BT474 versus StratRef were performed. Background subtraction and computation of ratios were carried out using GenePix 3.0 (Axon Instruments, Sunnyvale, CA). Background subtraction was performed by subtracting the median feature background intensity from median feature pixel intensity at each wavelength (635 nm for Cy5 and 532 nm for Cy3). The background subtracted values were then used to calculate the ratio of medians (Cy5/Cy3) which corresponds to the raw ratio value for each gene.

### Microarray Data analysis

Gene expression was analyzed with Cluster[25] using the average linkage metric and displayed using Treeview[26]. Genepix median of ratio values from the experiment were subjected to linear normalization in NOMAD[24], log-transformed (base 2) and filtered for genes where data were present in more than 90% of experiments. After linear normalization, log (base 2) transformation and hierarchical clustering, the cluster dataset was imported into the SAM software package. One class analysis was performed to identify genes representative of StratRef and genes representative of BT474 (with 2–4 fold differences in expression). Data was censored if more than one data value was flagged in each group to eliminate poor quality array data. Delta was chosen to limit the output gene list so that less than 1% predicted false positives would be included. Pearson correlation coefficients comparing microarray and QPCR gene expression measurements were made in Excel (Microsoft, Redmond, WA). Global Pearson correlation coefficients for microarrays were calculated using the statistical software package R:limma[27].

### Quantitative RT-PCR

cDNA was made from total RNA for both BT474 and StratRef, in 100-μL reactions using M-MLV reverse transcriptase and random hexamers incubated at 25°C for 10 min then 48°C for 30 min. Expression of each gene was analyzed using the 5' nuclease assay (real-time TaqMan RT-PCR; [28])with the ABI PRISM 7700 instrument (Applied Biosystems (ABI), Foster City, CA). QPCR was performed in triplicate technical replicates. Probe sequences and cycle conditions are available upon request. Relative expression levels were calculated compared to beta-glucuronidase as detailed previously [29,30].

### Statistical Analysis

The mean expression ratios for each of the 3 amplification techniques and for the total RNA method were calculated for each technique. Analysis A is defined as the 37 QPCR genes, Analysis B, the high intensity dataset, is defined as all genes that had a 635 Median Intensity OR a 532 Median Intensity > 1500. Analysis C, the unfiltered dataset, is defined as all of the genes on the microarray that had data present for 90% of the arrays. Sensitivity, specificity and percentage correct were calculated for each method in Analysis A using QPCR results as the gold standard. All pairs of correlation coefficients for Analysis B and C were used to perform intramethod and intermethod comparisons of mean correlation coefficients for all methods using expression results from microarrays of unamplified RNA as the gold standard.

## Results

**Figure 1:**
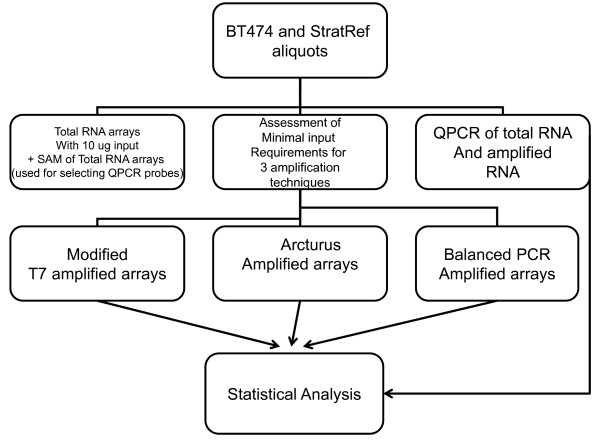
**Study Design – BT474 and Stratagene Universal Human Pooled Reference RNA were used as the substrate for these experiments**. 10 ug of total RNA from each were hybridized to microarrays and labeled "total RNA arrays". SAM analysis from these total RNA arrays were used to select QPCR genes in an unbiased fashion prior to performing any amplification reaction. Total RNA was serially diluted, amplified, and hybridized to cDNA microarrays. QPCR was performed on total RNA and amplified RNA. Statistical analyses included microarray vs microarray analysis as well as microarray vs QPCR analysis.

Figure 1 demonstrates a schematic of our study design. To validate our microarray results, we utilized quantitative RT-PCR (QPCR) for a panel of 37 genes. Unlike prior studies that used QPCR to validate expression of outliers – genes predominantly expressed in one RNA sample versus another – we selected QPCR primers to measure genes that are under-expressed, equivalent, or over-expressed in BT474 relative to StratRef total (unamplified) RNA (see Additional file 1 for graphical distribution of QPCR expression). Half of the genes were selected based on SAM[31] (Significance analysis of microarrays) analysis of total RNA BT474 expression relative to StratRef (2–4 fold under/over-expression) (Additional file 2 total RNA). The other half was selected to include genes that did not have a minimum of a 2-fold change in expression, which are genes considered to have low levels of expression on microarray analysis. Thus, our QPCR validation experiments were designed to determine whether fidelity of amplification was compromised without regard to the amplitude of the ratio of gene expression between two RNA samples. Our QPCR gene set covers a wide dynamic range of gene expression (including genes with equivalent expression in BT474 relative to StratRef) and was selected before amplifications were performed (see Additional file 3 for delta delta CTs).

### Lowest Input RNA Concentrations Required for Reproducible RNA Amplification

The lower limits of total RNA required for each method were defined as the lowest RNA input amount where amplification reactions consistently yielded sufficient product to permit analysis on cDNA microarrays and confirmatory functional assays. These were 500 pg for modified T7, 250 pg for Arcturus RiboAmp HS, and 500 pg for balanced PCR (Table 1). All 3 techniques had some failures to amplify low-input RNA, with successful input ranges depending on the technique tested. At or below the threshold of 500 pg, the Arcturus RiboAmp HS amplification method was more likely to produce a successful expression profile and also provided enough material for multiple hybridizations or other biological analyses.

### Assessment of Transcript Length

The size of the amplified products ranged from 100–4400 bases for all attempted amplifications. For the modified T7 method, after two rounds of amplification the product measured a mean of 3400 bases on the Agilent Bioanalyzer PicoChip. For Arcturus RiboAmp HS, after two rounds of linear amplification the product measured a mean of 3372 bases on the Agilent Bioanalyzer PicoChip. The Balanced PCR products were evaluated on a 1% Agarose gel and measured over 3500 bases (data not shown). Amplification reactions that did not yield the requisite 1.25 ug of RNA were considered unsuccessful; all successful reactions were validated with the Agilent Bioanalyzer and none required study exclusion on that basis.

### Pairwise correlation coefficients of microarray data

The Pearson's correlations and standard deviations for intra-method and inter-method comparisons for the high intensity genes are shown in Table 2. This data represents the 739 most intensely expressed genes, identified by a 635 Median Intensity or a 532 Median Intensity > 1500. The Pearson's correlations and standard deviations for intra-method and inter-method comparisons for the all unselected genes are shown in Table 3. This represents 17,001 genes found to be present as data points in >90% of the microarrays.

**Table 2:** The Pearson's correlations and standard deviations for intra-method and inter-method comparisons for 739 high intensity genes.

**Unamp A**	**Unamp A**																
**Unamp B**	0.78	**Unam p B**															
**Unamp C**	0.78	0.90	**Unamp C**														
**Mod T7 D**	0.72	0.79	0.78	Mod T7 D*													
**Mod T7 E**	0.72	0.80	0.77	0.89	**Mod T7 E**												
**Mod T7 F**	0.73	0.77	0.76	0.90	0.87	**Mod T7 F**											
**Mod T7 G**	0.73	0.77	0.76	0.90	0.86	0.94	**Mod T7 G**										
**Mod T7 H**	0.74	0.81	0.79	0.91	0.87	0.94	0.96	**Mod T7 H**									
**Mod T7 I**	0.78	0.80	0.79	0.90	0.86	0.94	0.95	0.95	**Mod T7 I**								
**Arcturus J**	0.71	0.78	0.77	0.89	0.87	0.89	0.91	0.92	0.90	**Arcturus J**							
**Arcturus K**	0.72	0.80	0.77	0.88	0.87	0.86	0.86	0.88	0.86	0.89	**Arcturus K**						
**Arcturus L**	0.73	0.79	0.79	0.88	0.85	0.86	0.88	0.89	0.88	0.88	0.86	**Arcturus L**					
**Arcturus M**	0.74	0.82	0.80	0.92	0.88	0.88	0.90	0.92	0.89	0.90	0.91	0.93	**Arcturus M**				
**Bal PCR N**	0.72	0.78	0.77	0.85	0.82	0.82	0.83	0.84	0.84	0.82	0.81	0.84	0.86	**Bal PCR N**			
**Bal PCR O**	0.71	0.77	0.75	0.85	0.82	0.80	0.80	0.82	0.82	0.80	0.81	0.82	0.85	0.93	**Bal PCR O**		
**Bal PCR P**	0.66	0.74	0.72	0.83	0.79	0.77	0.78	0.79	0.79	0.76	0.78	0.79	0.83	0.87	0.86	**Bal PCR P**	
**Bal PCR Q**	0.68	0.73	0.72	0.81	0.78	0.76	0.77	0.78	0.78	0.76	0.77	0.80	0.82	0.89	0.90	0.85	**Bal PCR Q**
																	
	**Intramethod**				**Intermethod Mean R2**												
	**Mean R**^**2**^	**SD**	**Range**	**Unamp**	**SD**	**Mod T7**	**SD**	**Arcturus**	**SD**	**Bal PCR**	**SD**						
**Unamp**	0.82	0.06	0.78–0.90			0.77	0.03	0.77	0.03	0.73	0.03						
**Mod T7**	0.91	0.03	0.86–0.96	0.77	0.03			0.88	0.02	0.81	0.03						
**Arcturus**	0.90	0.02	0.86–0.93	0.77	0.03	0.88	0.02			0.81	0.03						
**Bal PCR**	0.88	0.03	0.82–0.93	0.73	0.03	0.81	0.03	0.81	0.03								

**Table 3:** The Pearson's correlations and standard deviations for intra-method and inter-method comparisons for the unfiltered gene set (17,001 genes).

**Unamp A**	**Unamp A**															
**Unamp B**	0.59	**Unamp B**														
**Unamp C**	0.56	0.73	**Unam p C**													
**Mod T7 D**	0.36	0.30	0.30	Mod T7 D*												
**Mod T7 E**	0.25	0.17	0.14	0.46	**Mod T7 E**											
**Mod T7 F**	0.36	0.30	0.29	0.60	0.41	**Mod T7 F**										
**Mod T7 G**	0.37	0.30	0.28	0.60	0.40	0.65	**Mod T7 G**									
**Mod T7 H**	0.30	0.21	0.21	0.50	0.37	0.51	0.54	**Mod T7 H**								
**Mod T7 I**	0.38	0.32	0.28	0.58	0.38	0.63	0.66	0.50	**Mod T7 I**							
**Arcturus J**	0.32	0.30	0.26	0.60	0.40	0.57	0.58	0.46	0.60	**Arcturus J**						
**Arcturus K**	0.30	0.24	0.21	0.52	0.37	0.48	0.47	0.40	0.48	0.56	**Arcturus K**					
**Arcturus L**	0.24	0.16	0.14	0.39	0.33	0.40	0.39	0.35	0.37	0.40	0.38	**Arcturus L**				
**Arcturus M**	0.36	0.27	0.26	0.67	0.46	0.60	0.60	0.50	0.57	0.61	0.57	0.49	**Arcturus M**			
**Bal PCR N**	0.29	0.20	0.19	0.42	0.29	0.35	0.37	0.31	0.38	0.37	0.37	0.41	0.36	**Bal PCR N**		
**Bal PCR O**	0.27	0.18	0.17	0.37	0.26	0.30	0.30	0.30	0.31	0.30	0.29	0.28	0.38	0.49	**Bal PCR O**	
**Bal PCR P**	0.33	0.25	0.22	0.48	0.31	0.39	0.38	0.34	0.38	0.36	0.39	0.34	0.50	0.56	0.49	**Bal PCR P**
**Bal PCR Q**	0.25	0.11	0.10	0.36	0.27	0.27	0.28	0.28	0.27	0.27	0.29	0.31	0.40	0.49	0.50	0.53
																
	**Intramethod**				**Intermethod Mean R2**											
	**Mean R**^**2**^	**SD**	**Range**	**Unamp**	**SD**	**Mod T7**	**SD**	**Arcturus**	**SD**	**Bal PCR**	**SD**					
**Unamp**	0.63	0.07	0.56–0.73			0.28	0.07	0. 26	0.06	0.21	0.07					
**Mod T7**	0.52	0.10	0.14–0.66	0.28	0.07			0.48	0.10	0.33	0.05					
**Arcturus**	0.50	0.09	0.14–0.61	0.26	0.06	0.48	0.10			0.35	0.06					
**Bal PCR**	0.51	0.03	0.10–0.56	0.21	0.07	0.33	0.05	0.35	0.06							

Table 4 shows a summary of the true and false calls, sensitivity and specificity of each amplification technique compared to the QPCR results. Table 5 presents a summary of the true and false calls, sensitivity and specificity of each method for the 739 most highly expressed genes using unamplified RNA as the gold standard. Table 6 shows a summary of the same results for the 17,001 genes, again using unamplified RNA as the gold standard.

**Table 4:** Statistical analysis of microarray versus QPCR results (37 genes)

	**Total 10 ug**	**Baugh 500 pg**	**Baugh 1 ng**	**Baugh 10 ng**	**Arcturus 250 pg**	**Arcturus 500 pg**	**Arcturus 1 ng**	**BalPCR 0.667 ng**	**BalPCR 3.33 ng**
**True Positives**	18	14	13	14	15	16	15	18	17
**False Positives**	3	1	1	2	1	1	0	2	3
**True Negatives**	15	17	17	16	17	17	18	16	15
**False Negatives**	1	5	6	5	4	3	4	1	2
**Sensitivity**	94.74%	73.68%	68.42%	73.68%	78.95%	84.21%	78.95%	94.74%	89.47%
**Specificity**	83.33%	94.44%	94.44%	88.89%	94.44%	94.44%	100.00%	88.89%	83.33%
**Total**	**37**	**37**	**37**	**37**	**37**	**37**	**37**	**37**	**37**
									
**% Correct**	89.2%	83.8%	81.1%	81.1%	86.5%	89.2%	89.2%	91.9%	86.5%

**Table 5:** Statistical analysis of high intensity gene set (739 genes)

	**Baugh 500 pg**	**Baugh 1 ng**	**Baugh 10 ng**	**Arcturus 250 pg**	**Arcturus 500 pg**	**Arcturus 1 ng**	**BalPCR 0.667 ng**	**BalPCR 3.33 ng**
**True Positives**	210	187	197	246	188	216	172	187
**False Positives**	150	95	127	274	123	161	93	144
**True Negatives**	332	387	304	208	328	319	389	338
**False Negatives**	47	70	48	11	58	41	85	70
**Sensitivity**	81.7%	72.8%	80.4%	95.7%	76.4%	84.0%	66.9%	72.8%
**Specificity**	68.9%	80.3%	70.5%	43.2%	72.7%	66.5%	80.7%	70.1%
**Blanks**	0	0	63	0	42	2	0	0
**Total**	**739**	**739**	**739**	**739**	**739**	**739**	**739**	**739**
								
**% Correct**	73.3%	77.7%	67.8%	61.4%	69.8%	72.4%	75.9%	71.0%

**Table 6:** Statistical analysis of unfiltered gene set (17,001 genes)

	**Baugh 500 pg**	**Baugh 1 ng**	**Baugh 10 ng**	**Arcturus 250 pg**	**Arcturus 500 pg**	**Arcturus 1 ng**	**BalPCR 0.667 ng**	**BalPCR 3.33 ng**
**True Positives**	3072	2454	2786	3366	3306	3297	2816	3373
**False Positives**	2801	1785	1976	3373	3716	3598	3202	4219
**True Negatives**	7448	8465	7882	6875	6190	6621	7047	6031
**False Negatives**	3679	4297	3818	3385	3276	3445	3934	3378
**Sensitivity**	45.5%	36.4%	42.2%	49.9%	50.2%	48.9%	41.7%	50.0%
**Specificity**	72.7%	82.6%	80.0%	67.1%	62.5%	64.8%	68.8%	58.8%
**Blanks**	0	0	539	0	513	40	0	0
**Total**	**17000**	**17001**	**17001**	**16999**	**17001**	**17001**	**16999**	**17001**
								
**% Correct**	61.9%	64.2%	62.7%	60.2%	55.9%	58.3%	58.0%	55.3%

### Validation of Microarray Data

QPCR of amplified RNA was correlated with the QPCR of total RNA for all methods with R2 of 0.87 for modified T7, R2 0.86 for Arcturus RiboAmp HS and R2 0.75 for balanced PCR.

When dealing with clinical samples, microarray results are often validated with QPCR; therefore techniques that demonstrate a low false expression result (FER) by this type of analysis are very desirable. We defined a FER as number of discordant values divided by number of genes analyzed for expression ratio by microarray compared to QPCR using unamplified total RNA. Both array and QPCR measurements were normalized to levels of β-glucuronidase. As shown in Table 7, Balanced PCR showed a mean percent FER of 11.3%, Arcturus RiboAmp HS showed 13.5%, and modified T7 showed 14.6%. The FER rates for each method were independent of input RNA level by ANOVA (p = 0.39).

**Table 7:** Rate of false expression microarray results based on QPCR gold standard by amplification technique

**Method**	**Quantitiy of Input**	**# False**	**% FER**
**Modified T7**	500 pg	5	13.5
	500 pg	5	13.5
	500 pg	6	19
	1 ng	6	19
	1 ng	5	13.5
	Mean	5.4	14.6
**Arcturus**	250 pg	6	19
	250 pg	6	19
	500 pg	4	10.8
	1 ng	4	10.8
	Mean	5	13.5
**Balanced PCR**	500 pg	1	2.7
	667 pg	5	13.5
	667 pg	4	10.8
	3.3 ng	5	13.5
	3.3 ng	6	19
	Mean	4.2	11.3

When we compared QPCR of amplified to total RNA for each method (in an effort to control for the potential bias of each individual array hybridization), the FER was 0% (0/21 genes) for Arcturus, 4.5% (1/22 genes) for modified T7 and 15% (3/19 genes) for Balanced PCR.

## Discussion

Not surprisingly, the unfiltered arrays showed poorer overall correlation even between replicate arrays than did the analysis filtered for high intensity spots. Even the unamplified arrays had correlation coefficients somewhat lower than expected when array data was unfiltered, indicating that the filtration process removes the effect of background and nonspecific hybridization on the cDNA arrays. The filtration process did not change the individual expression ratios but rather focused the analysis on genes in which the hybridization gave a particularly strong signal, a strategy that seems logical when attempting to work with very rare clinical specimens to ensure a lower false call rate. Other groups have reported analysis of total RNA arrays with input as high as 20–40 ug per microarray, which may have improved the results for our total RNA arrays[32] That being said, in real world situations researchers will not have abundant amounts of clinically relevant total RNA to use for comparisons to low-input RNA samples. Confirmation of array results will require reliance on functional assays such as QPCR to validate results, which is why we emphasized the QPCR component of our analysis. All three methods tested showed comparable accuracy rates that were within the range of previous reports based on amplification from picogram amounts of total RNA[17]. However, our study also determined the minimal input requirements of each of the 3 amplification methods. Table 1 shows the threshold for each technique – for example, above 250 pg, the Arcturus method worked reproducibly, while the modified T7 and balanced PCR techniques required approximately 1 ng of total RNA.

RNA amplification technologies serve translational clinical research well. Linear amplification already has enabled examination of gene expression in clinical core needle biopsies[1], surgical biopsies[33], fine needle aspirates[34] and even single human cells[35]. Our analysis of the high intensity spots demonstrates that amplification is reproducible and highly correlated with QPCR measurements using sub-nanogram input RNA samples. These results clearly demonstrate that data-processing has a marked impact on expression array results, particularly when working with very low-input samples.

While each method was able to provide data in the sub-nanogram range, certain methods are advantageous over others in terms of lower limit of RNA that can reliably be amplified, cost per reaction and the number of days required for processing of samples. The Arcturus RiboAmp HS method was more reliable at generating expression arrays at the threshold of below a nanogram of total RNA. For modified T7 and balanced PCR, a nanogram of total RNA will ensure reliability. The Arcturus amplification procedure was able to produce very substantial amounts of nucleic acids from just 250 pg of total RNA and therefore should be considered more reliable than the other two methods at lower input thresholds. Caretti et al. performed a comparison of amplifications of a colon biopsy subjected to laser capture microdissection with purification of an estimated 1 nanogram of RNA per specimen; they compared the two cycle Arcturus OA and the one cycle Nugen amplification and found that the Arcturus method showed the lowest variance and highest correlation[36]. However, our data is distinct in that we examined the Arcturus Ribo Amp HS kit, which permits RNA amplification from lower input samples than the Arcturus OA or Nugen kits. In addition, our study provides detailed comparisons of amplified RNA to both total RNA and QPCR, which is not available in the study by Caretti et al. Wilhelm et al compared Arcturus Ribo Amp (a less sensitive kit than Ribo Amp HS) to SMART PCR (Clontech) and concluded that SMART is preferable when working with less than 200 ng of total RNA[37]. Our study is distinct in that we show that IVT based amplification (Ribo Amp HS) is more reliable than PCR based amplification at the sub-nanogram input level. Clontech's SMART PCR is marketed for use at the nanogram low-input range, not the sub-nanogram range, which is why we did not include SMART PCR in our comparison.

Shearstone et al performed a comparison of their laboratory's novel IVT amplification, termed BIIB, to Arturus Ribo Amp HS, Nugen Ovation, Affymetrix One and Affymetrix Two Cycle[38]. Their method, BIIB, proved to be capable of successful amplification with good reproducibility from 50 pg of total RNA, and could amplify from lower input amounts of RNA than commercially available kits. They emphasized that T7 based linear amplification has advantages over PCR based amplification because T7 accurately retains the transcript stoichiometry of the original sample. Our study is similar to that of Shearstone et al in the design by which they use a reference RNA for the baseline from which to determine which of several amplification techniques performs the best at low inputs of RNA. Our study is distinct in that we found that Arcturus Ribo Amp HS had acceptable correlation coefficients in the 250 pg range when attention was focused to the high intensity spots for data analysis. Our study provides additional insight on the impact of data processing on microarray results at low input of total RNA, which was not the focus of Shearstone et al. Although BIIB performed well in their hands, Arcturus Ribo Amp HS is currently commercially available, kit based, and takes less time to perform than BIIB. Further studies are needed to compare these two methods and perhaps improve upon them with the ultimate goal being consistent successful amplification from even a single cell.

Our paper describes a platform independent measure, the FER, useful in comparing amplification methods based on QPCR versus microarray. FER was calculated based on QPCR of total RNA rather than amplified RNA, since there was not sufficient amplified product available for performing QPCR on all 37 primer probe pairs. In addition, QPCR of amplified RNA is biased towards recapitulating the results of the microarray experiment due to truncation of the RNA products.

Each of the three amplification techniques yielded fairly consistent expression results within the constraints of each technique's input threshold of total RNA, both on microarray analysis and when compared to QPCR. Based on these excellent correlations, it is feasible to reproducibly perform high fidelity amplifications by a variety of techniques when starting with sub-nanogram input quantities of total RNA. However, when attempting expression arrays analysis from less than 500 pg input, linear amplification with Arcturus RiboAmp HS was more successful than the other methods studied.

Below 1 ng, the modified T7 method could not reproducibly amplify such that insufficient RNA was typically generated for even a single microarray hybridization. Only by performing multiple attempts at amplification were we able to achieve successful amplified product with this technique. While we were successful in hybridizing 3 arrays with this method at 500 pg we do not recommend this method below 1 ng of input total RNA as several operators quite experienced with this method could not repeat these results. One drawback of this technique is the greater length of time involved (3 days) compared to other amplification reactions (2 days) and the relative complexity of the protocol.

Arcturus RiboAmp HS was able to provide expression array data at a lower input concentration than any of the other tested methods and we were able to use smaller amounts than the manufacturer's recommended minimum sample input of 500 pg total RNA. Below 250 pg, this method typically failed to amplify in our hands (although the manufacturer validated the Arcturus method to a threshold of 100 picograms). This likely represents a theoretical limit of 25 cells total RNA content (for laser capture microdissection more would be required because of fractionation of cellular material), unless specialized tissues that bear more RNA such as oocytes[35] are examined. Since each somatic mammalian cell is thought to have approximately 10 pg of total RNA, the Arcturus method is especially promising for limiting clinical samples, suggesting that clinical samples comprised of approximately 25 cells could be routinely analyzed with Arcturus, or 100 cells with modified T7 or balanced PCR, with profound implications for correlative science studies using gene expression profiling that have previously not considered using such small quantities. It is somewhat concerning, however, that the % FER was observed to increase from 10.8% at 500 pg to 19% at 250 pg in our study. This is a potential area of future investigation as scientists seek to push the lower limit of input RNA required

Balanced PCR is a promising technique for the amplification of low-input quantities of RNA. It maintains a high degree of accuracy with an input as low as 667 pg of RNA (FER 10.8–13.5%). While theoretical concern exists regarding the accuracy of logarithmic amplification methods, this method overcomes the potential problem by stopping the PCR reaction before the logarithmic phase of the PCR curve. This method had the lowest cost per reaction and also required the least amount of technician time compared to the other methods. In addition, it has been recently demonstrated that the same balanced-PCR protocol used for cDNA amplification may also be used for the unbiased amplification of whole genomic DNA followed by array-CGH analysis[21]. However, several iterations were required for experienced personnel to learn to successfully perform balanced PCR.

These results have certain important limitations to consider. First, hybridizations were carried out sequentially over a period of several months rather than all at the same time. It is recognized that arrays that are hybridized together under identical conditions are more similar to each other than arrays hybridized on separate occasions. Additionally, no dye swap experiments were performed. The rationale for this is that since a standardized universal reference (StratRef) was employed, it has been demonstrated that this mitigates the effect of potential experimental bias introduced by separate hybridization reactions and even permits the comparisons of array data between members of the research community[15]. Use of a universal reference may in some cases have advantages over dye swap experiments. [15]

Each laboratory will have to weigh their decision on which amplification technique is most suitable based on factors including amount of starting input total RNA, cost per reaction, technician time, and experience/comfort level with the techniques. Laboratories that routinely work with samples in excess of 1 ng starting material should focus on cost-savings as each of the methods tested proved to be reliable above this threshold. Balanced PCR could be further optimized to include amino-allyl-dUTP incorporation in the PCR reaction. This would facilitate indirect Cy dye labeling, which would reduce the labeling cost for this method.

It is important to ascertain the linearity of a chosen method at the low input range before going on to work with precious clinical specimens. Each of the 3 tested methods performed surprisingly accurately when amplifying from low inputs of total RNA based on microarray analysis validated with QPCR of 37 genes.

## Conclusion

We have demonstrated that it is feasible to reliably and accurately perform expression profiling from sub-nanogram quantities of total RNA. These methods will likely enable exciting new directions for molecular analysis of samples previously considered to be of insufficient quantity of total RNA for expression profiling. The data processing and filtration of microarray results is of fundamental importance when attempting analysis of amplification reactions from sub-nanogram input amounts of total RNA.

## Abbreviations

(QPCR): quantitative real-time PCR; (SMART PCR): switching mechanism of 5'end of RNA template PCR; (StratRef): Stratagene Universal Human Reference RNA.

## Authors' contributions

JEL developed the study design, carried out the preparation of RNA, the total RNA array hybridizations, the Arcturus and modified T7 RNA amplifications and hybridizations, quantification and assessment of transcript integrity, coordinated efforts with the laboratory of GMM, participated in data analysis, drafted the manuscript and performed critical revisions. MJM participated in data analysis and critical revision of the manuscript. JS performed the QPCR and provided the BT474 cell line. GMM and GW performed the balanced PCR reactions. LJE participated in study design and conception. JWP participated in study design and conception. CMH and his laboratory provided the modified T7 protocol, conducted gene expression array quality control and assisted with data analysis. CMH participated in study design.

## Supplementary Material

Additional file 1 **Table S2**. QPCR genes. Click here for file

Additional file 2 **Figure S1**: Dynamic Range of QPCR Probes for BT474 Versus StratRef. The delta delta CT of our QPCR probes covered a dynamic range of negative 21 to positive 9 and were selected without bias towards any of the amplification techniques. Click here for file

Additional file 3 **Table S1-8**. SAM Analysis. Click here for file
