# Supplementary material for: A comparison of RNA amplification techniques at sub-nanogram input concentration
Source: BMC Genomics. 2009 Jul 20;10:326. doi: 10.1186/1471-2164-10-326 (PMC2724417; doi:10.1186/1471-2164-10-326)
Supplement: Additional file 1 — Table S2. QPCR genes. [file 1471-2164-10-326-S1.pdf]

Gene	Accession Number	**Delta Delta of Total RNA**
THSB2	H38240	-21.17
APOB	H93837	-15.57
PRG1	AA278759	-13.02
IGF2	N54596	-12.36
MMP3	W51794	-12.24
TF	H69531	-11.2
CAV1	AA055835	-10.71
FN1	R62612	-8.49
FGF7	AA009609	-6.97
TGFB	AA233809	-4.61
TIMP2	AA486280	-3.42
LEUP	W49781	-2.94
TM4SF1	AA487893	-2.55
PGCP	AA460833	-1.84
CDK6	CDK6	-0.69
ANXA1	H63077	-0.24
MADH	R82176	-0.23
ELK1	AA844141	0.03
CASP2	R37937	0.24
TRAF3	AA504259	0.47
MMD	AA454572	0.55
DFF	AA487452	1.08
GRP	AA026118	1.89
NUP88	AA479888	2.85
RAE1	AA504128	3.19
CDH1	H97778	4.08
CCNG2	AA489752	4.11
IGFBP5	H08560	4.94
CLA4	AA430665	5.15
MGP	AA155913	5.19
PIP5K2B	H80359	5.56
PRLR	R63647	5.75
v-erbb	AA443351	6.81
CEACAM6	AA054073	7.76
APM2	AA478298	8.24
TCN1	AA155695	9.22
GUSB	N34827	N/A
