# Supplementary figures and images for: A comparison of RNA amplification techniques at sub-nanogram input concentration
Source: BMC Genomics. 2009 Jul 20;10:326. doi: 10.1186/1471-2164-10-326 (PMC2724417; doi:10.1186/1471-2164-10-326)

Dynamic Range of QPCR probes for BT474 Versus StratRef

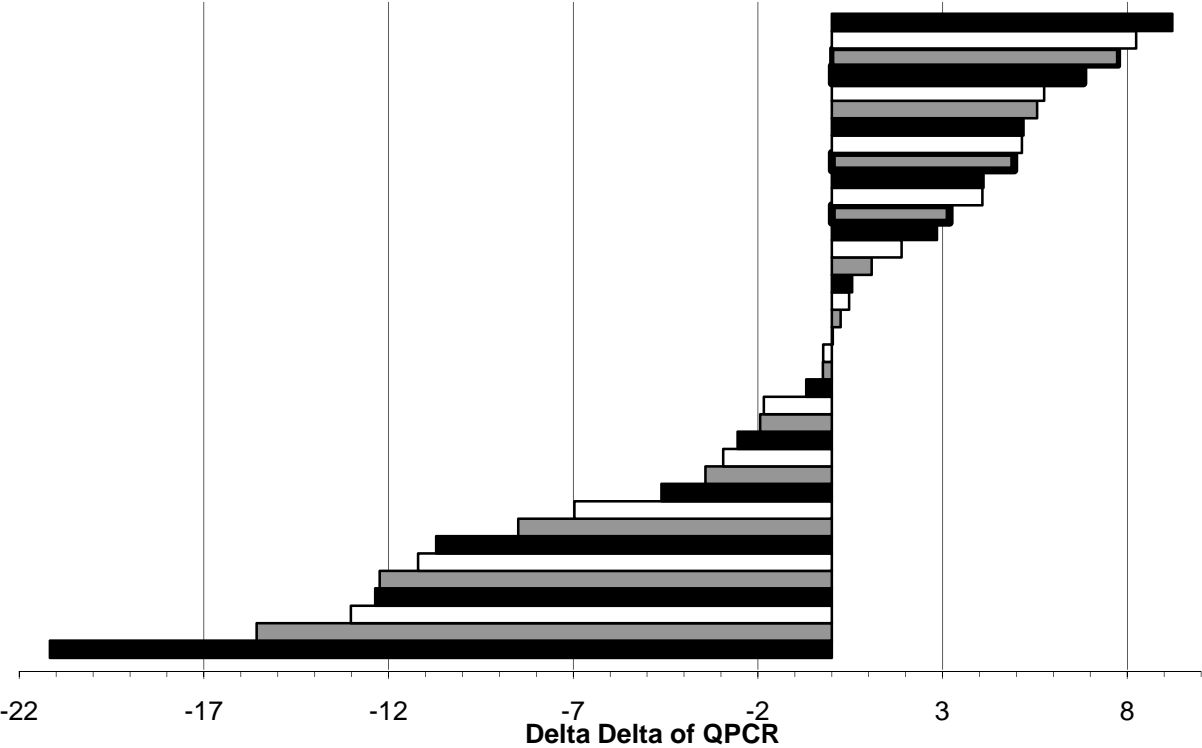

Supplement: Additional file 2 — Figure S1: Dynamic Range of QPCR Probes for BT474 Versus StratRef. The delta delta CT of our QPCR probes covered a dynamic range of negative 21 to positive 9 and were selected without bias towards any of the amplification techniques. [file 1471-2164-10-326-S2.pdf]
