# Supplementary material for: A comparison of RNA amplification techniques at sub-nanogram input concentration
Source: BMC Genomics. 2009 Jul 20;10:326. doi: 10.1186/1471-2164-10-326 (PMC2724417; doi:10.1186/1471-2164-10-326)

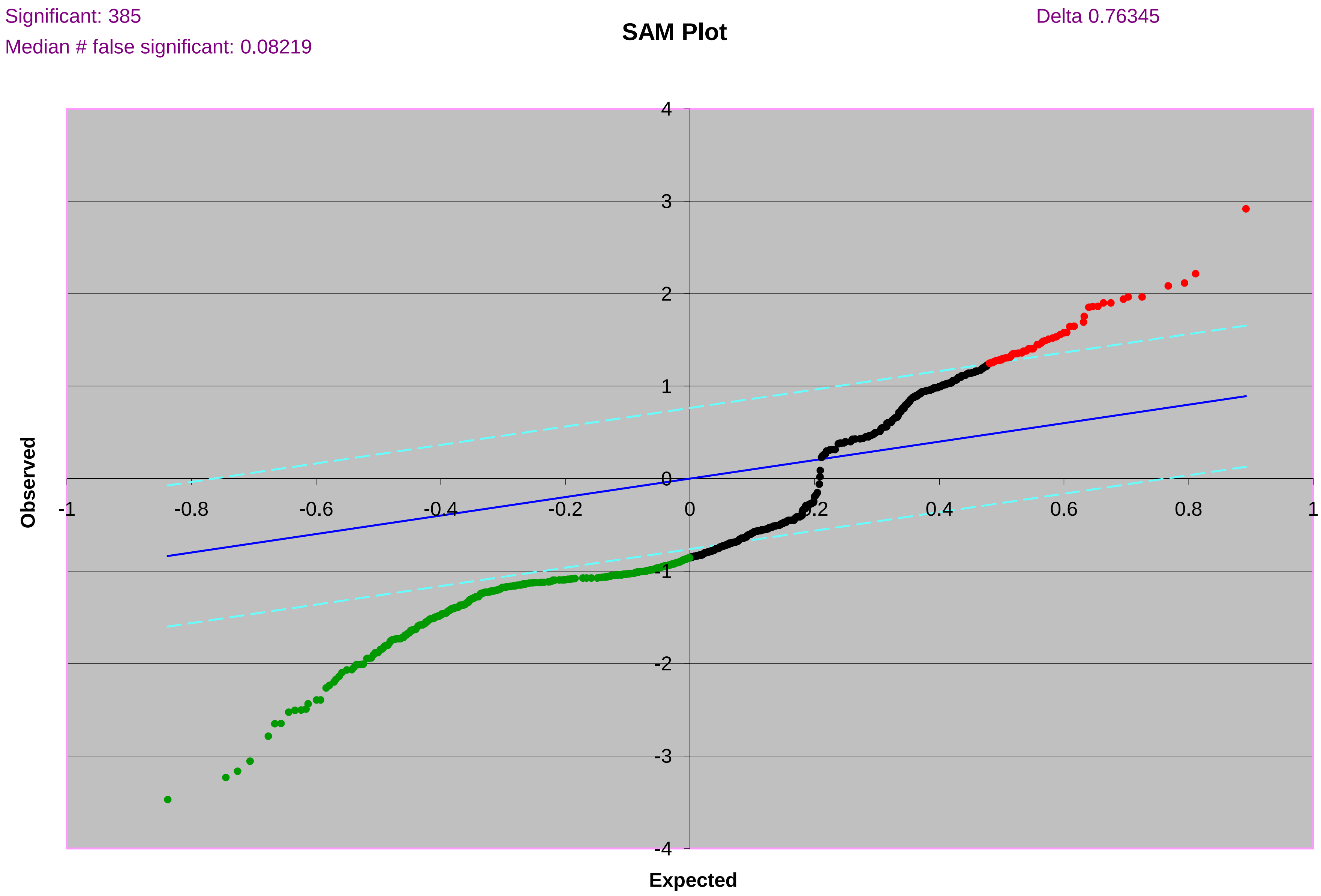

Significant: 385
Median # false significant: 0.08219
SAM Plot
Delta 0.76345
Observed
Expected
4
3
2
1
0
-1
-2
-3
-4
-1
-0.8
-0.6
-0.4
-0.2
0
0.2
0.4
0.6
0.8
1

Significant Genes List

Input Parameters

Imputation Engine
Data Type
Number of Permutations
Blocked Permutation?
RNG Seed
Delta
(Upper Cutoff, Lower Cutoff)

Computed Quantities

Computed Exchangeability Factor S0
S0 percentile
False Significant Number (Median, 90 percentile)
False Discovery Rate (Median, 90 percentile)
Pi0Hat

55 Positive Significant Genes

Row	Gene Name	Gene ID
553	W72838::ESTs	W72838
555	missing90	missing90
649	H20138::RAB6, member RAS oncogene family	H20138
616	AA478298::adipose specific 2	AA478298
605	AA155695::transcobalamin I (vitamin B12 binding protein, R binder family)	AA155695
621	R63647::prolactin receptor	R63647
586	AA029597::bone morphogenetic protein 7 (osteogenic protein 1)	AA029597
606	AA054073::carcinoembryonic antigen-related cell adhesion molecule 6 (non-specific cross reacting antigen)	AA054073
561	N48258::ESTs	N48258
580	W73473::bone morphogenetic protein 7 (osteogenic protein 1)	W73473
629	R68997::ESTs	R68997
533	AA055768::ESTs	AA055768
614	H13688::UDP-N-acetyl-alpha-D-galactosamine:polypeptide N-acetylgalactosaminyltransferase 3 (GalNAc-T3)	H13688
637	AA496149::3-hydroxy-3-methylglutaryl-Coenzyme A synthase 2 (mitochondrial)	AA496149
633	AA218915::EST	AA218915
636	R70505::purinergic receptor P2Y, G-protein coupled, 2	R70505
607	N50681::ESTs	N50681
581	T95747::pregnancy specific beta-1-glycoprotein 1	T95747
570	AA485052::proteasome (prosome, macropain) 26S subunit, non-ATPase, 3	AA485052
563	N30553::pregnancy specific beta-1-glycoprotein 4	N30553
559	W91882::EST	W91882
669	N74131::Human secretory protein (P1.B) mRNA, complete cds	N74131
641	R76554::calmodulin 1 (phosphorylase kinase, delta)	R76554
599	AA464421::zinc finger protein 144 (Mel-18)	AA464421
608	AA453783::Homo sapiens mRNA; cDNA DKFZp564B1264 (from clone DKFZp564B1264)	AA453783
578	H97778::cadherin 1, E-cadherin (epithelial)	H97778
619	H95976::ESTs	H95976
571	AA455291::ESTs	AA455291
603	T56316::nerve growth factor, beta polypeptide	T56316
615	W51985::pregnancy specific beta-1-glycoprotein 11	W51985
648	AA456598::ESTs	AA456598
536	R73909::pregnancy specific beta-1-glycoprotein 11	R73909
631	AA495846::ras homolog gene family, member B	AA495846
558	W37780::ESTs	W37780
592	AA151245::ESTs	AA151245
584	AA136565::ESTs	AA136565
587	T77883::EST	T77883
632	AA477298::branched chain keto acid dehydrogenase E1, alpha polypeptide (maple syrup urine disease)	AA477298
611	H01197::ESTs	H01197
668	AA453404::PPAR binding protein	AA453404
582	H80359::phosphatidylinositol-4-phosphate 5-kinase, type II, beta	H80359
537	AA708301::ESTs	AA708301
528	AA262080::solute carrier family 12 (sodium/potassium/chloride transporters), member 2	AA262080
639	N90806::ESTs	N90806
595	R32952::S100 calcium-binding protein P	R32952
589	W47576::ESTs	W47576
541	AA418081::ESTs	AA418081
560	H62594::tyrosine 3-monooxygenase/tryptophan 5-monooxygenase activation protein, beta polypeptide	H62594
590	AA489569::keratin 7	AA489569
617	H62594::tyrosine 3-monooxygenase/tryptophan 5-monooxygenase activation protein, beta polypeptide	H62594
609	AA046424::ESTs, Weakly similar to HYPOTHETICAL PROTEIN ZAP128 [H.sapiens]	AA046424
597	H53703::growth factor receptor-bound protein 7	H53703
554	R76229::ESTs, Highly similar to CGI-52 protein [H.sapiens]	R76229
569	AA464250::keratin 19	AA464250
576	AA504128::RAE1 (RNA export 1, S.pombe) homolog	AA504128

330 Negative Significant Genes

Row	Gene Name	Gene ID
233	N68719::ESTs	N68719
230	H79534::hemoglobin, epsilon 1	H79534
266	T90857::ESTs	T90857
208	R62612::fibronectin 1	R62612
164	H93837::apolipoprotein B (including Ag(x) antigen)	H93837
302	R62612::fibronectin 1	R62612
173	W56586::ESTs	W56586
254	H59861::thrombomodulin	H59861
171	AA598601::insulin-like growth factor binding protein 3	AA59860
318	H95960::secreted protein, acidic, cysteine-rich (osteonectin)	H95960
219	R95691::CAG repeat domain	R95691
137	AA478542::A kinase (PRKA) anchor protein (gravin) 12	AA47854
247	H69531::transferrin	H69531
180	N54596::insulin-like growth factor 2 (somatomedin A)	N54596
270	R11698::ubiquinol-cytochrome c reductase hinge protein	R11698
136	N54596::insulin-like growth factor 2 (somatomedin A)	N54596
207	N59721::CAG repeat domain	N59721
255	R60343::5' nucleotidase (CD73)	R60343
294	T67053::Human rearranged immunoglobulin lambda light chain mRNA	T67053
176	H38240::thrombospondin 2	H38240
260	H96654::ESTs, Weakly similar to gene pp21 protein [H.sapiens]	H96654
306	R45941::protein tyrosine phosphatase, receptor type, N	R45941
214	N63943::lysozyme (renal amyloidosis)	N63943
501	AA620437::Homo sapiens mRNA; cDNA DKFZp566E183 (from clone DKFZp566E183)	AA62043
234	T62547::insulin-like growth factor 2 receptor	T62547
223	R02346::small nuclear ribonucleoprotein 70kD polypeptide (RNP antigen)	R02346
342	AA043133::solute carrier family 16 (monocarboxylic acid transporters), member 1	AA04313
248	R17717::cadherin 13, H-cadherin (heart)	R17717
414	R01937::EST	R01937
245	AA453969::lactate dehydrogenase C	AA45396
212	H99415::A kinase (PRKA) anchor protein 2	H99415
143	AA233809::transforming growth factor, beta 2	AA23380
141	AA039370::Homo sapiens transcriptional enhancer factor (TEF1) DNA, complete CDS	AA03937
335	H68848::apolipoprotein H (beta-2-glycoprotein I)	H68848
221	AA282906::CD44 antigen (homing function and Indian blood group system)	AA28290
222	W81128::Homo sapiens mRNA; cDNA DKFZp586H0919 (from clone DKFZp586H0919)	W81128
283	H09461::putative gene product	H09461
483	missing51	missing51
175	AA055835::caveolin 1, caveolae protein, 22kD	AA05583
284	AA775872::glypican 3	AA77587
420	AA482593::ESTs	AA48259
477	W45275::CD44 antigen (homing function and Indian blood group system)	W45275
381	AA399473::tissue factor pathway inhibitor 2	AA39947
206	N91921::T-cell receptor, beta cluster	N91921
152	AA633993::cell division cycle 10 (homologous to CDC10 of S. cerevisiae)	AA63399
182	AA487812::vimentin	AA48781
240	AA086475::cullin 5	AA08647
224	R93124::aldo-keto reductase family 1, member C1 (dihydrodiol dehydrogenase 1; 20-alpha (3-alpha)-hydroxysteroid dehydrogenase)	R93124
149	AA112105::farnesyltransferase, CAAX box, alpha	AA11210
156	AA683077::ribosomal protein L37	AA68307
261	T97889::solute carrier family 2 (facilitated glucose transporter), member 3	T97889
349	AA055946::CD3D antigen, delta polypeptide (TiT3 complex)	AA05594
187	T56948::Human mRNA for KIAA0099 gene, complete cds	T56948
326	AA279072::inositol polyphosphate phosphatase-like 1	AA27907
217	H86554::ceruloplasmin (ferroxidase)	H86554
200	AA282134::glutaminyl-peptide cyclotransferase (glutaminyl cyclase)	AA28213
465	N26285::fibronectin 1	N26285
253	T89996::FOS-like antigen-1	T89996
190	H26271::ESTs	H26271
153	AA436479::ESTs, Weakly similar to Grb14 [H.sapiens]	AA43647
158	R92216::ESTs, Weakly similar to putative p150 [H.sapiens]	R92216
322	AA678160::ESTs	AA67816
201	H23978::general transcription factor IIB	H23978
415	AA400292::ESTs	AA40029
198	Missing157	Missing15
280	N34436::v-maf musculoaponeurotic fibrosarcoma (avian) oncogene homolog	N34436
268	T77595::hexabrachion (tenascin C, cytotactin)	T77595
329	AA046525::Homo sapiens, alpha-1 (VI) collagen	AA04652
272	H64380::ESTs	H64380
250	H10068::Homo sapiens clone 24466 mRNA sequence	H10068
277	SSH121e1	SSH121e
144	AA150532::keratin 6B	AA15053
183	T53220::fatty acid binding protein 1, liver	T53220
205	AA775447::alpha-2-macroglobulin	AA77544
344	W49672::wingless-type MMTV integration site family, member 5A	W49672
276	AA099153::tissue inhibitor of metalloproteinase 3 (Sorsby fundus dystrophy, pseudoinflammatory)	AA09915
151	N73836::ESTs	N73836

238 H14231::ESTs	H14231
169 AA706738::ESTs	AA70673
330 AA862371::interferon-inducible	AA86237
210 R62603::collagen, type VI, alpha 3	R62603
518 AA775616::secreted phosphoprotein 1 (osteopontin, bone sialoprotein I, early T-lymphocyte activation 1)	AA77561
278 R37411::Homo sapiens guanine deaminase (GDA), mRNA	R37411
202 AA284693::transcription factor AP-4 (activating enhancer-binding protein 4)	AA28469
313 H17550::ESTs	H17550
495 AA488626::ubiquitin-like 1 (sentrin)	AA48862
399 T98352::Homo sapiens clone 24483 unknown mRNA, parital cds	T98352
327 AA449333::proteasome (prosome, macropain) subunit, alpha type, 4	AA44933
251 W69134::ESTs	W69134
471 AA700876::orosomucoid 1	AA70087
292 AA682293::phenylalanine hydroxylase	AA68229
474 AA194983::tumor necrosis factor receptor superfamily, member 11b (osteoprotegerin)	AA19498
271 AA063573::ESTs	AA06357
305 AA425947::regulated in glioma	AA42594
147 AA227594::mal, T-cell differentiation protein	AA22759
298 missing226	missing22
237 N67039::ESTs	N67039
341 H87471::kynureninase (L-kynurenine hydrolase)	H87471
274 AA043501::v-maf musculoaponeurotic fibrosarcoma (avian) oncogene homolog	AA04350
179 H15662::KIAA0291 protein	H15662
155 AA496878::ESTs, Weakly similar to FK506-binding protein [H.sapiens]	AA49687
407 T68892::secreted frizzled-related protein 1	T68892
379 AA292074::ubiquitin-conjugating enzyme E2L 6	AA29207
166 R97220::ESTs	R97220
246 R22977::moesin	R22977
218 H80685::P311 protein	H80685
199 AA136707::procollagen-lysine, 2-oxoglutarate 5-dioxygenase (lysine hydroxylase) 2	AA13670
263 H58644::ESTs	H58644
249 R10382::protein C inhibitor (plasminogen activator inhibitor III)	R10382
347 AA447528::homolog of yeast (S. cerevisiae) ufd2	AA44752
216 AA258396::pleckstrin homology-like domain, family A, member 1	AA25839
384 AA633751::palmitoyl-protein thioesterase 2	AA63375
220 R92227::ESTs, Weakly similar to beta-TrCP protein E3RS-IkappaB [M.musculus]	R92227
161 N93505::transmembrane 4 superfamily member 2	N93505
312 H99816::procollagen-lysine, 2-oxoglutarate 5-dioxygenase (lysine hydroxylase) 2	H99816
145 T82817::FOS-like antigen-1	T82817
512 AA485371::bone marrow stromal cell antigen 2	AA48537
297 AA102526::interleukin 8	AA10252
148 N21633::ESTs, Highly similar to NESTIN [H.sapiens]	N21633
282 W86431::protein C inhibitor (plasminogen activator inhibitor III)	W86431
359 R42177::KIAA0759 protein	R42177
299 N47524::ESTs, Highly similar to mitogen-induced [M.musculus]	N47524
135 AA454562::ESTs	AA45456
242 W73144::lymphocyte cytosolic protein 1 (L-plastin)	W73144
418 AA633569::aldehyde dehydrogenase 10 (fatty aldehyde dehydrogenase)	AA63356
293 AA159620::ecotropic viral integration site 2B	AA15962
189 T48949::Rhesus blood group-associated glycoprotein	T48949
378 AA456474::apolipoprotein C-II	AA45647
493 AA701860::follistatin	AA70186
482 N52362::Homo sapiens mRNA; cDNA DKFZp586E1624 (from clone DKFZp586E1624)	N52362
307 W74668::glycophorin C (Gerbich blood group)	W74668
343 AA011681::DiGeorge syndrome gene D	AA01168
256 AA256231::Gardner-Rasheed feline sarcoma viral (v-fgr) oncogene homolog	AA25623
337 AA487846::ESTs	AA48784
236 H15718::AXL receptor tyrosine kinase	H15718
150 AA418392::ESTs	AA41839
388 AA431795::ESTs	AA43179
410 R43605::KIAA0293 protein	R43605
336 AA490238::mitogen inducible 2	AA49023
301 AA431571::ESTs	AA43157
496 R06634::inter-alpha (globulin) inhibitor, H2 polypeptide	R06634
498 AA863383::pim-2 oncogene	AA86338
311 H23235::platelet-derived growth factor receptor, alpha polypeptide	H23235
195 N39240::ESTs	N39240
229 AA487893::transmembrane 4 superfamily member 1	AA48789
403 R09069::glucan (1,4-alpha-), branching enzyme 1 (glycogen branching enzyme, Andersen disease, glycogen storage disease type IV)	R09069
333 H57052::ESTs	H57052
286 AA479781::radixin	AA47978
186 N40975::EST	N40975
134 N77198::ESTs	N77198
300 T80932::ESTs	T80932
332 AA406546::Homo sapiens mRNA; cDNA DKFZp564F053 (from clone DKFZp564F053)	AA40654
204 AA477165::radixin	AA47716
324 AA291577::interferon-stimulated transcription factor 3, gamma (48kD)	AA29157
196 AA293671::CD8 antigen, beta polypeptide 1 (p37)	AA29367

225	AA173428::ESTs	AA173428
172	R59724::ESTs	R59724
227	AA150263::ESTs	AA150263
209	AA291163::glutaredoxin (thioltransferase)	AA291163
340	R33755::glutathione S-transferase pi	R33755
241	AA486280::tissue inhibitor of metalloproteinase 2	AA486280
296	N74889::ESTs	N74889
285	AA620379::KIAA0878 protein	AA620379
133	AA459868::ubiquitin-conjugating enzyme E2E 3 (homologous to yeast UBC4/5)	AA459868
281	AA489636::ESTs	AA489636
413	R52339::Homo sapiens clone 25114 mRNA sequence	R52339
235	AA668470::regulator of G-protein signalling 5	AA668470
138	AA598794::connective tissue growth factor	AA598794
257	R88506::ESTs, Weakly similar to SRrp129 [H.sapiens]	R88506
363	R37566::ESTs	R37566
239	AA173755::ESTs	AA173755
211	AA233079::insulin-like growth factor binding protein 1	AA233079
486	AA599094::KIAA0878 protein	AA599094
383	N67770::silver (mouse homolog) like	N67770
303	AA884897::ELL-RELATED RNA POLYMERASE II, ELONGATION FACTOR	AA884897
409	AA447978::retinaldehyde dehydrogenase 2	AA447978
481	H91680::phosphorylase, glycogen; liver (Hers disease, glycogen storage disease type VI)	H91680
197	H09748::ESTs	H09748
226	H20717::ESTs	H20717
252	N73091::ribosomal protein S23	N73091
459	AA406551::solute carrier family 2 (facilitated glucose transporter), member 3	AA406551
162	H95348::ESTs	H95348
275	H10192::ESTs	H10192
323	N26562::melan-A	N26562
416	AA443119::Homo sapiens mRNA; cDNA DKFZp586L2123 (from clone DKFZp586L2123)	AA443119
258	AA936799::matrix metalloproteinase 2 (gelatinase A, 72kD gelatinase, 72kD type IV collagenase)	AA936799
310	R92801::ESTs	R92801
449	N51499::A kinase (PRKA) anchor protein 2	N51499
315	T83821::ESTs, Weakly similar to KIAA0638 protein [H.sapiens]	T83821
243	AA486082::serum/glucocorticoid regulated kinase	AA486082
203	W01240::membrane protein, palmitoylated 1 (55kD)	W01240
146	R66057::ESTs, Weakly similar to envelope protein [H.sapiens]	R66057
269	R71124::ESTs	R71124
139	AA458653::Homo sapiens mRNA for GS3955, complete cds	AA458653
475	AA490777::glutathione S-transferase theta 2	AA490777
264	AA456394::four and a half LIM domains 1	AA456394
193	AA127069::DKFZP586E1621 protein	AA127069
441	AA775257::integral membrane protein 2A	AA775257
505	AA704688::ESTs	AA704688
419	R09561::decay accelerating factor for complement (CD55, Cromer blood group system)	R09561
168	H05445::growth associated protein 43	H05445
228	H57309::slug (chicken homolog), zinc finger protein	H57309
464	AA148524::ESTs	AA148524
412	AA461065::mercaptopyruvate sulfurtransferase	AA461065
265	W01011::SA (rat hypertension-associated) homolog	W01011
92	AA137180::EST	AA137180
259	AA620462::ESTs, Weakly similar to TRANSFORMATION-SENSITIVE PROTEIN IEF SSP 3521 [H.sapiens]	AA620462
321	AA598817::preferentially expressed antigen in melanoma	AA598817
194	T53298::insulin-like growth factor binding protein 7	T53298
485	H46553::transcription factor 8 (represses interleukin 2 expression)	H46553
181	AA001614::insulin receptor	AA001614
348	AA228130::PC4 and SFRS1 interacting protein 1	AA228130
262	AA488609::nucleoporin 88kD	AA488609
398	AA436565::ESTs	AA436565
287	R26163::ESTs, Weakly similar to !!!! ALU CLASS A WARNING ENTRY !!!! [H.sapiens]	R26163
288	N20407::coagulation factor II (thrombin) receptor	N20407
479	AA496283::Thy-1 cell surface antigen	AA496283
174	AA169469::pyruvate dehydrogenase kinase, isoenzyme 4	AA169469
476	AA858026::protein C inhibitor (plasminogen activator inhibitor III)	AA858026
360	AA490172::collagen, type I, alpha 2	AA490172
167	AA287196::tetraspan 3	AA287196
140	AA457544::EST	AA457544
177	R76553::Homo sapiens a disintegrin-like and metalloprotease (reprolysin type) with thrombospondin type 1 motif, 1 (ADAMTS1), mRNA	R76553
160	R56916::Homo sapiens mRNA; cDNA DKFZp586F071 (from clone DKFZp586F071)	R56916
338	AA131466::ESTs	AA131466
408	AA172056::ESTs	AA172056
159	AA609421::Homo sapiens BAC clone RG298G08 from 7p15-p21	AA609421
191	R76614::ESTs	R76614
372	AA670200::postmeiotic segregation increased 2-like 12	AA670200
289	AA496005::albumin	AA496005
170	AA418750::Homo sapiens clone 683 unknown mRNA, complete sequence	AA418750
215	N62244::TAR (HIV) RNA-binding protein 1	N62244
345	R11047::ESTs	R11047

244	AA455067::synuclein, alpha (non A4 component of amyloid precursor)	AA45506
316	T89391::caveolin 2	T89391
346	T95274::ESTs	T95274
470	AA284668::plasminogen activator, urokinase	AA28466
290	AA164712::ESTs, Weakly similar to TRANSFORMATION-SENSITIVE PROTEIN IEF SSP 3521 [H.sapiens]	AA16471
314	R78530::ESTs, Highly similar to deduced protein product shows significant homology to coactosin from Dictyostelium discoideum [H.sapiens]	R78530
308	AA496955::ESTs	AA49695
185	H73724::cyclin-dependent kinase 6	H73724
462	R10378::fibrinogen-like 1	R10378
319	N22980::FYN oncogene related to SRC, FGR, YES	N22980
213	N62638::ESTs	N62638
374	N52517::EST	N52517
489	W70343::lysyl oxidase	W70343
331	AA447746::ESTs	AA44774
178	H63077::annexin A1	H63077
267	AA156749::ESTs	AA15674
231	AA449738::erythrocyte membrane protein band 4.1-like 2	AA44973
325	AA171784::ESTs	AA17178
320	N32295::alpha2,3-sialyltransferase	N32295
163	R83836::v-yes-1 Yamaguchi sarcoma viral related oncogene homolog	R83836
291	AA432268::ESTs	AA43226
511	AA683578::adenosine deaminase	AA68357
232	R98628::EST	R98628
356	N24076::KIAA0704 protein	N24076
157	T95151::ESTs	T95151
339	AA704222::ESTs	AA70422
423	R38539::fibroblast growth factor 2 (basic)	R38539
355	H73479::ESTs, Weakly similar to EPIDERMAL GROWTH FACTOR RECEPTOR KINASE SUBSTRATE EPS8 [H.sapiens]	H73479
411	N77263::Human beta-type globin pseudogene	N77263
142	AA487192::ESTs, Moderately similar to !!!! ALU SUBFAMILY SQ WARNING ENTRY !!!! [H.sapiens]	AA48719
334	AA644657::major histocompatibility complex, class I, A	AA64465
366	H68922::integrin, alpha 1	H68922
309	AA487561::ESTs, Highly similar to RAS-RELATED PROTEIN RAB-1A [M.musculus]	AA48756
351	N74623::insulin-like growth factor 2 (somatomedin A)	N74623
461	H99676::collagen, type VI, alpha 1	H99676
386	AA420992::ESTs	AA42099
506	AA495981::ESTs	AA49598
328	AA497044::ESTs, Highly similar to 3-7 gene product [H.sapiens]	AA49704
406	AA398356::ESTs	AA39835
417	AA853966::Human mRNA for KIAA0298 gene, complete cds	AA85396
184	H13424::Putative prostate cancer tumor suppressor	H13424
154	N26665::Human (lambda) DNA for immunogloblin light chain	N26665
442	N21338::ESTs, Weakly similar to Enkephalinase analogue polypeptide [H.sapiens]	N21338
499	N25883::ESTs	N25883
440	W69995::ESTs	W69995
165	AA701448::ESTs, Weakly similar to T15B7.2 [C.elegans]	AA70144
192	N64734::ESTs	N64734
188	H97146::ESTs, Highly similar to G protein-coupled receptor kinase 6, splice variant B [H.sapiens]	H97146
396	AA410591::met proto-oncogene (hepatocyte growth factor receptor)	AA41059
421	AA436459::nuclear factor I/X (CCAAT-binding transcription factor)	AA43645
468	N26108::ESTs	N26108
478	AA485449::ESTs, Weakly similar to small GTP-binding protein Rab36 [H.sapiens]	AA48544
380	N94616::laminin, alpha 4	N94616
401	AA419608::ESTs, Weakly similar to !!!! ALU SUBFAMILY J WARNING ENTRY !!!! [H.sapiens]	AA41960
317	AA456376::coagulation factor II (thrombin) receptor	AA45637
438	AA058533::ESTs	AA05853
424	T96083::proteasome (prosome, macropain) subunit, alpha type, 4	T96083
273	AA430367::cystathionine-beta-synthase	AA43036
304	AA418007::ESTs, Highly similar to mitogen-induced [M.musculus]	AA41800
358	AA032221::Homo sapiens PAC clone DJ1121E10 from 7q21.1-q2	AA03222
99	T74567::ESTs	T74567
371	W80701::ESTs, Weakly similar to HERV-E envelope glycoprotein [H.sapiens]	W80701
507	AA458814::ESTs	AA45881
460	R94808::ESTs	R94808
514	N98485::forkhead box F2	N98485
402	R49597::ESTs	R49597
448	N64741::slug (chicken homolog), zinc finger protein	N64741
94	H27864::secretogranin II (chromogranin C)	H27864
123	AA151413::EST	AA15141
458	T64625::esterase D/formylglutathione hydrolase	T64625
279	R40481::ESTs, Weakly similar to KIAA0872 protein [H.sapiens]	R40481
295	AA406354::ESTs, Weakly similar to similar to ERG-3 like protein [C.elegans]	AA40635
404	H94469::ESTs, Weakly similar to cDNA EST EMBL:D36107 comes from this gene [C.elegans]	H94469
422	R66310::peptidylglycine alpha-amidating monooxygenase	R66310
466	AA704802::ESTs	AA70480
425	R26756::ESTs	R26756
405	N42770::tyrosinase (oculocutaneous albinism IA)	N42770
395	W73874::cathepsin L	W73874

62	R33402::ESTs	R33402
370	R52965::ESTs	R52965
400	AA452145::ESTs, Weakly similar to PHOSPHOLIPID HYDROPEROXIDE GLUTATHIONE PEROXIDASE [H.sapiens]	AA45214[illegible]
387	AA427667::T-cell receptor, alpha (V,D,J,C)	AA42766[illegible]
382	AA490048::EST	AA49004[illegible]
469	H94043::DKFZP586I1419 protein	H94043
491	AA457501::ESTs	AA45750[illegible]
487	N63635::pim-1 oncogene	N63635
397	AA598653::osteoblast specific factor 2 (fasciclin I-like)	AA59865[illegible]
377	N33322::ESTs	N33322
484	W73810::epithelial membrane protein 3	W73810
492	H77697::ESTs	H77697
109	AA449361::ring finger protein 13	AA44936[illegible]
467	R38459::ESTs	R38459
450	N66120::ESTs, Highly similar to PROTEIN-TYROSINE PHOSPHATASE DELTA PRECURSOR [H.sapiens]	N66120
502	AA620401::ESTs	AA62040[illegible]
508	R28660::ESTs	R28660
490	N66144::FYN oncogene related to SRC, FGR, YES	N66144
433	W92703::ESTs	W92703

Significant: 385

Median # false significant: 0.08219

Delta 0.76345

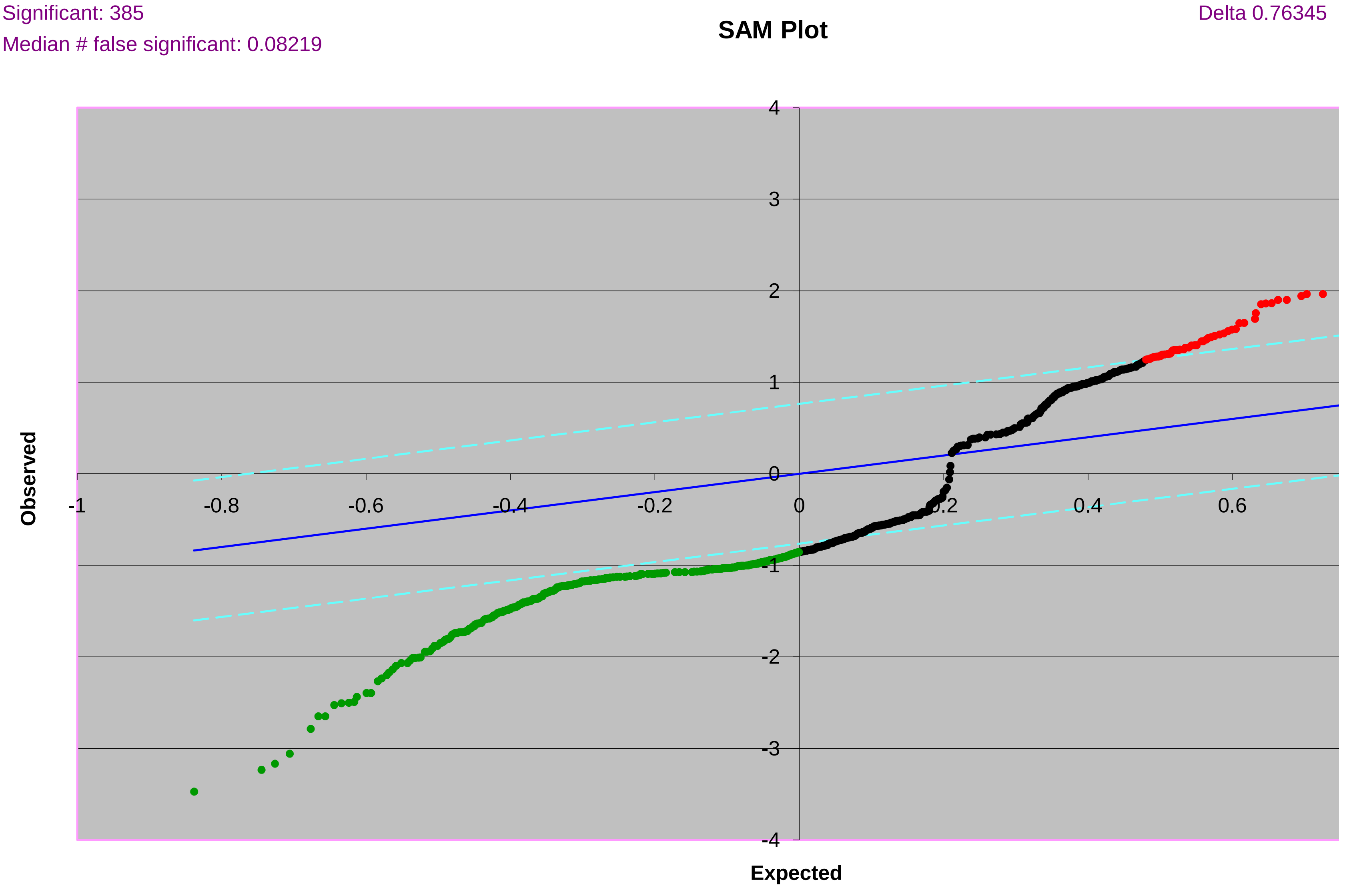

Row Average Imputer
One class Response
24
FALSE
1234567
0.76345
(1.24861, -0.85642)

1.957794763
1
(0.08219, 0.57534)
(0.02135, 0.14944)
0.08219

Gene ID	Score(d)	Numerator(r)	Denominator(s+s0)	q-value (%)
W72838	2.91773979	5.7435	1.968475743	0.01670565
missing90	2.217947173	4.3775	1.973671895	0.01670565
H20138	2.114505235	4.611	2.180651967	0.01670565
AA478298	2.083743498	4.3785	2.101266305	0.01670565
AA155695	1.964286379	4.1445	2.109926559	0.01670565
R63647	1.963788911	4.1015	2.088564599	0.01670565
AA029597	1.942540495	3.8945	2.00484881	0.01670565
AA054073	1.901203756	4.035	2.12233959	0.01670565
N48258	1.899847968	3.759	1.978579373	0.01670565
W73473	1.864211321	3.738	2.005137485	0.01670565
R68997	1.860386606	3.856	2.072687466	0.01670565
AA055768	1.852279931	3.944	2.129267793	0.01670565
H13688	1.755834047	3.6175	2.060274436	0.01670565
AA496149	1.693061316	3.691	2.180074616	0.01670565
AA218915	1.648256749	3.58	2.171991713	0.01670565
R70505	1.644492033	3.5685	2.169970987	0.01670565
N50681	1.581877165	3.2865	2.077594944	0.01670565
T95747	1.575204678	3.1535	2.001962058	0.01670565
AA485052	1.557712021	3.0915	1.98464155	0.01670565
N30553	1.53498961	3.0145	1.963856941	0.01670565
W91882	1.522120584	3.009	1.976847322	0.01670565
N74131	1.506965851	3.4145	2.265811131	0.01670565
R76554	1.492640473	3.1485	2.109349208	0.01670565
AA464421	1.483907412	2.996	2.018993891	0.01670565
AA453783	1.467656009	3.0475	2.076440243	0.01670565
H97778	1.447991426	2.8495	1.967898393	0.01670565
H95976	1.447290322	2.9885	2.064893238	0.01670565
AA455291	1.405409708	2.801	1.993013129	0.01670565
T56316	1.404029021	2.932	2.088275924	0.01670565
W51985	1.403301156	2.8685	2.044108628	0.01670565
AA456598	1.38010114	2.89	2.094049426	0.01670565
R73909	1.377287629	2.869	2.083079771	0.01670565
AA495840	1.359182028	2.887	2.12407164	0.01670565
W37780	1.357925792	2.6605	1.959238138	0.01670565
AA151249	1.353009247	2.72	2.010333637	0.01670565
AA136565	1.35147909	2.706	2.002250734	0.01670565
T77883	1.351054847	2.6915	1.992147104	0.01670565
AA477298	1.348503529	2.8795	2.135329971	0.01670565
H01197	1.332141942	2.7515	2.065470588	0.01670565
AA453404	1.312562824	2.96	2.255130151	0.01670565
H80359	1.30914613	2.6065	1.990992403	0.01670565
AA708301	1.305153766	2.721	2.084811822	0.01670565
AA262080	1.301754622	2.6955	2.07066674	0.01670565
N90806	1.294806323	2.7155	2.097224853	0.01670565
R32952	1.29404948	2.594	2.004560135	0.01670565
W47576	1.286022695	2.569	1.997631931	0.01670565
AA418081	1.281974784	2.592	2.021880643	0.01670565
H62594	1.281229123	2.518	1.965300316	0.01670565
AA489569	1.279795892	2.544	1.987816977	0.01670565
H62594	1.276849472	2.6255	2.056232984	0.01670565
AA046424	1.270652722	2.6355	2.074130842	0.01670565
H53703	1.261555658	2.5285	2.004271459	0.01670565
R76229	1.254593987	2.47	1.968764418	0.01670565
AA464250	1.250544242	2.4595	1.966743692	0.01670565
AA504128	1.248605383	2.4665	1.975403946	0.01670565

Gene ID	Score(d)	Numerator(r)	Denominator(s+s0)	q-value (%)
N68719	-3.47157164	-6.971	2.008024236	0.01670565
H79534	-3.23278216	-6.5335	2.021014617	0.01670565
T90857	-3.1661171	-6.2635	1.978290697	0.01670565
R62612	-3.05635723	-6.199	2.028231496	0.01670565
H93837	-2.78668983	-5.932	2.128690443	0.01670565
R62612	-2.65008296	-5.86	2.211251531	0.01670565
W56586	-2.64916483	-5.8725	2.216736359	0.01670565
H59861	-2.52623021	-5.0545	2.000807358	0.01670565
AA59860	-2.50654694	-5.4565	2.17689919	0.01670565
H95960	-2.50195626	-5.2905	2.114545361	0.01670565
R95691	-2.49217775	-4.895	1.964145616	0.01670565
AA47854	-2.43644082	-5.012	2.057099009	0.01670565
H69531	-2.39432776	-4.7505	1.9840642	0.01670565
N54596	-2.39381524	-5.2415	2.189600896	0.01670565
R11698	-2.26522255	-4.4865	1.980600098	0.01670565
N54596	-2.23414383	-4.5965	2.057387684	0.01670565
N59721	-2.19937819	-4.4145	2.007158211	0.01670565
R60343	-2.17115528	-4.366	2.010910988	0.01670565
T67053	-2.13805051	-4.65	2.174878464	0.01670565
H38240	-2.09761623	-4.5445	2.166506885	0.01670565
H96654	-2.0688172	-4.0545	1.959815489	0.01670565
R45941	-2.06714904	-4.3675	2.11281331	0.01670565
N63943	-2.04102902	-4.0295	1.974249245	0.01670565
AA62043	-2.01525912	-5.038	2.499926666	0.01670565
T62547	-2.01305905	-4.026	1.999941332	0.01670565
R02346	-2.00965313	-4.0685	2.024478719	0.01670565
AA04313	-2.00701779	-4.1605	2.072976142	0.01670565
R17717	-1.94495079	-3.91	2.010333637	0.01670565
R01937	-1.94424899	-4.3155	2.21962311	0.01670565
AA45396	-1.93878026	-3.8545	1.988105652	0.01670565
H99415	-1.90678417	-3.743	1.962990915	0.01670565
AA23380	-1.8813358	-3.8185	2.029674871	0.01670565
AA03937	-1.88095459	-3.8275	2.034871024	0.01670565
H68848	-1.84832217	-3.7675	2.038335125	0.01670565
AA28290	-1.84495634	-3.693	2.001673383	0.01670565
W81128	-1.83444536	-3.7085	2.021591968	0.01670565
H09461	-1.82835302	-3.949	2.159867357	0.01670565
missing51	-1.81181337	-4.066	2.244160496	0.01670565
AA05583	-1.8044958	-3.811	2.111947285	0.01670565
AA77587	-1.80171113	-3.9055	2.167661586	0.01670565
AA48259	-1.78463848	-4.0045	2.243871821	0.01670565
W45275	-1.75590884	-4.1215	2.347217519	0.01670565
AA39947	-1.74659845	-3.962	2.268409208	0.01670565
N91921	-1.74065161	-3.4405	1.976558647	0.01670565
AA63399	-1.74036974	-3.5565	2.043531278	0.01670565
AA48781	-1.7401201	-3.666	2.106751132	0.01670565
AA08647	-1.73405521	-3.4605	1.995611205	0.01670565
R93124	-1.73255256	-3.453	1.993013129	0.01670565
AA11210	-1.73254646	-3.4955	2.017550516	0.01670565
AA68307	-1.73253802	-3.556	2.052480207	0.01670565
T97889	-1.72796999	-3.3875	1.960392839	0.01670565
AA05594	-1.71944816	-4.2295	2.459800822	0.01670565
T56948	-1.71725511	-3.598	2.095204127	0.01670565
AA27907	-1.70416104	-3.544	2.07961567	0.01670565
H86554	-1.69333172	-3.3675	1.988683002	0.01670565
AA28213	-1.68934716	-3.6395	2.154382529	0.01670565
N26285	-1.6710861	-3.8375	2.296410696	0.01670565
T89996	-1.66695698	-3.3035	1.981754799	0.01670565
H26271	-1.64579568	-3.4345	2.086832548	0.01670565
AA43647	-1.63757088	-3.3625	2.053346232	0.01670565
R92216	-1.63683464	-3.335	2.0374691	0.01670565
AA67816	-1.63240253	-3.391	2.077306269	0.01670565
H23978	-1.62832957	-3.4695	2.130711168	0.01670565
AA40029	-1.59405336	-3.4885	2.188446195	0.01670565
Missing15	-1.58973565	-3.3735	2.122050914	0.01670565
N34436	-1.58588421	-3.396	2.141392148	0.01670565
T77595	-1.58356025	-3.114	1.966455017	0.01670565
AA04652	-1.5813749	-3.285	2.077306269	0.01670565
H64380	-1.58101074	-3.381	2.138505397	0.01670565
H10068	-1.57014879	-3.1325	1.995033855	0.01670565
SSH121e	-1.56458924	-3.3915	2.167661586	0.01670565
AA15053	-1.54986103	-3.105	2.003405434	0.01670565
T53220	-1.54721025	-3.2645	2.109926559	0.01670565
AA77544	-1.54183515	-3.2585	2.11339066	0.01670565
W49672	-1.52154152	-3.108	2.042665252	0.01670565
AA09915	-1.51618005	-3.28	2.163331459	0.01670565
N73836	-1.51467595	-3.052	2.014952439	0.01670565

H14231	-1.51270267	-3.0275	2.001384708	0.01670565
AA70673!	-1.51084381	-3.0735	2.034293673	0.01670565
AA86237:	-1.50413654	-3.082	2.049016105	0.01670565
R62603	-1.49799675	-2.9505	1.969630443	0.01670565
AA77561(	-1.49736529	-3.758	2.50974162	0.01670565
R37411	-1.49100533	-3.2165	2.157269281	0.01670565
AA28469:	-1.4885436	-3.1665	2.127247067	0.01670565
H17550	-1.48831838	-3.0715	2.063738537	0.01670565
AA48862(	-1.47895221	-3.4885	2.358764525	0.01670565
T98352	-1.47653274	-3.184	2.156403255	0.01670565
AA44933:	-1.47507286	-3.0365	2.058542385	0.01670565
W69134	-1.46496004	-2.9015	1.980600098	0.01670565
AA70087(	-1.46078413	-3.3495	2.292946594	0.01670565
AA68229:	-1.45975919	-3.0665	2.100688954	0.01670565
AA19498:	-1.45844177	-3.3155	2.273316685	0.01670565
AA06357:	-1.45584167	-2.8515	1.958660788	0.01670565
AA42594	-1.45243938	-3.018	2.077883619	0.01670565
AA22759	-1.44238366	-2.908	2.01610714	0.01670565
missing22	-1.43262457	-2.976	2.077306269	0.01670565
N67039	-1.43000108	-2.869	2.006292185	0.01670565
H87471	-1.42288747	-2.8995	2.037757775	0.01670565
AA04350	-1.41755856	-3.0245	2.13359792	0.01670565
H15662	-1.41638995	-2.948	2.08134772	0.01670565
AA49687!	-1.4104122	-2.881	2.042665252	0.01670565
T68892	-1.40563756	-3.0015	2.135329971	0.01670565
AA29207	-1.40488315	-3.1175	2.21904576	0.01670565
R97220	-1.4037536	-2.852	2.031695597	0.01670565
R22977	-1.39524198	-2.7465	1.968475743	0.01670565
H80685	-1.39320995	-2.7425	1.968475743	0.01670565
AA13670	-1.39297083	-2.9455	2.114545361	0.01670565
H58644	-1.39107792	-2.7375	1.967898393	0.01670565
R10382	-1.3829553	-2.781	2.010910988	0.01670565
AA44752!	-1.37326885	-2.775	2.020725942	0.01670565
AA25839(	-1.37325798	-2.706	1.970496469	0.01670565
AA63375	-1.36729017	-3.04	2.223375887	0.01670565
R92227	-1.36706023	-2.6855	1.964434291	0.01670565
N93505	-1.36704801	-2.794	2.043819953	0.01670565
H99816	-1.36677194	-2.825	2.066913964	0.01670565
T82817	-1.36541595	-2.741	2.007446886	0.01670565
AA48537	-1.36465244	-3.2575	2.387054688	0.01670565
AA10252(	-1.3639297	-2.827	2.072687466	0.01670565
N21633	-1.36187178	-2.7445	2.015241115	0.01670565
W86431	-1.35645415	-2.835	2.090007974	0.01670565
R42177	-1.34668773	-3.2325	2.400333744	0.01670565
N47524	-1.34177718	-2.7745	2.067779989	0.01670565
AA45456:	-1.34073563	-2.717	2.026499445	0.01670565
W73144	-1.33794649	-2.665	1.991858429	0.01670565
AA63356!	-1.32597191	-2.88	2.171991713	0.01670565
AA15962(	-1.31294798	-2.7255	2.075862893	0.01670565
T48949	-1.30659207	-2.681	2.051902857	0.01670565
AA45647	-1.30375527	-2.8675	2.19941585	0.01670565
AA70186(	-1.29969385	-2.973	2.287461767	0.01670565
N52362	-1.29949925	-2.818	2.168527611	0.01670565
W74668	-1.29800292	-2.6645	2.052768882	0.01670565
AA01168	-1.29576846	-2.6255	2.02621077	0.01670565
AA25623	-1.29172048	-2.5375	1.964434291	0.01670565
AA48784(	-1.28714803	-2.5575	1.986950951	0.01670565
H15718	-1.28492426	-2.5605	1.992724454	0.01670565
AA41839:	-1.27947465	-2.567	2.006292185	0.01670565
AA43179!	-1.27824418	-2.8815	2.254264126	0.01670565
R43605	-1.27806868	-2.697	2.110215234	0.01670565
AA49023!	-1.27719399	-2.558	2.002828084	0.01670565
AA43157	-1.27670785	-2.63	2.05998576	0.01670565
R06634	-1.27180391	-2.907	2.285729716	0.01670565
AA86338:	-1.2659891	-2.9065	2.295833345	0.01670565
H23235	-1.25646256	-2.597	2.066913964	0.01670565
N39240	-1.24255295	-2.5765	2.073553492	0.01670565
AA48789:	-1.24073572	-2.442	1.968187068	0.01670565
R09069	-1.24031167	-2.6585	2.143412874	0.01670565
H57052	-1.23853359	-2.482	2.003982784	0.01670565
AA47978	-1.2325086	-2.5635	2.079904345	0.01670565
N40975	-1.22908012	-2.5365	2.063738537	0.01670565
N77198	-1.22831578	-2.4945	2.030829572	0.01670565
T80932	-1.22726636	-2.5115	2.046418029	0.01670565
AA40654(	-1.22620874	-2.475	2.018416541	0.01670565
AA47716!	-1.22208741	-2.545	2.082502421	0.01670565
AA29157	-1.21699035	-2.4715	2.030829572	0.01670565
AA29367	-1.21629529	-2.521	2.072687466	0.01670565

AA173428	-1.21599198	-2.4105	1.982332149	0.01670565
R59724	-1.21528217	-2.4975	2.055078283	0.01670565
AA150263	-1.21406839	-2.4165	1.990415053	0.01670565
AA291163	-1.21157736	-2.4	1.980888774	0.01670565
R33755	-1.20852906	-2.4285	2.009467612	0.01670565
AA486280	-1.20631292	-2.384	1.976269971	0.01670565
N74889	-1.20318033	-2.5025	2.079904345	0.01670565
AA620379	-1.20171914	-2.5175	2.094915452	0.01670565
AA459868	-1.19702586	-2.4275	2.027942821	0.01670565
AA489630	-1.19245036	-2.506	2.10155498	0.01670565
R52339	-1.17982333	-2.489	2.109637884	0.01670565
AA668470	-1.17520267	-2.3215	1.975403946	0.01670565
AA598794	-1.17487362	-2.349	1.999363982	0.01670565
R88506	-1.17266857	-2.306	1.966455017	0.01670565
R37566	-1.17116823	-2.7855	2.378394434	0.01670565
AA173755	-1.16930525	-2.3085	1.974249245	0.01670565
AA233079	-1.16926691	-2.3145	1.979445398	0.01670565
AA599094	-1.16479136	-2.501	2.147165651	0.01670565
N67770	-1.16471061	-2.521	2.164486159	0.01670565
AA884897	-1.16136258	-2.4115	2.076440243	0.01670565
AA447978	-1.16044532	-2.424	2.088853274	0.01670565
H91680	-1.15797032	-2.479	2.140814798	0.01670565
H09748	-1.15228687	-2.387	2.071532766	0.01670565
H20717	-1.14955542	-2.2715	1.975981296	0.01670565
N73091	-1.14879371	-2.269	1.975115271	0.01670565
AA406551	-1.14707647	-2.4825	2.164197484	0.01670565
H95348	-1.14007466	-2.2995	2.016973165	0.01670565
H10192	-1.1397137	-2.3955	2.101843655	0.01670565
N26562	-1.13941629	-2.311	2.028231496	0.01670565
AA443119	-1.13870462	-2.4085	2.115122711	0.01670565
AA936799	-1.13769464	-2.23	1.960104164	0.01670565
R92801	-1.13410405	-2.331	2.055366958	0.01670565
N51499	-1.13013669	-2.8295	2.503679442	0.01670565
T83821	-1.12627663	-2.285	2.028808846	0.01670565
AA486082	-1.12463284	-2.2255	1.978868048	0.01670565
W01240	-1.12444883	-2.3355	2.077017593	0.01670565
R66057	-1.12182031	-2.2575	2.012354363	0.01670565
R71124	-1.12004933	-2.198	1.962413565	0.01670565
AA458653	-1.1162216	-2.2385	2.00542616	0.01670565
AA490777	-1.11583604	-2.45	2.195663074	0.01670565
AA456394	-1.11357049	-2.184	1.961258864	0.01670565
AA127069	-1.10485263	-2.246	2.032850298	0.01670565
AA775257	-1.09797044	-2.8225	2.570652074	0.01670565
AA704688	-1.09730487	-2.479	2.259171603	0.01670565
R09561	-1.09527087	-2.3375	2.13417527	0.01670565
H05445	-1.0951951	-2.227	2.033427648	0.01670565
H57309	-1.09282659	-2.1695	1.985218901	0.01670565
AA148524	-1.09244632	-2.369	2.168527611	0.01670565
AA461069	-1.08832647	-2.2655	2.081636396	0.01670565
W01011	-1.08760216	-2.145	1.97222852	0.01670565
AA137180	-1.08460102	-2.6535	2.446521766	0.01670565
AA620462	-1.08319969	-2.1285	1.965011641	0.01670565
AA598817	-1.07920089	-2.187	2.026499445	0.01670565
T53298	-1.07552616	-2.2165	2.060851786	0.01670565
H46553	-1.07423195	-2.265	2.108483183	0.01670565
AA001614	-1.07377419	-2.207	2.055366958	0.01670565
AA228130	-1.0736308	-2.141	1.99416783	0.01670565
AA488609	-1.06848448	-2.0965	1.96212489	0.01670565
AA436569	-1.06793201	-2.359	2.20894213	0.01670565
R26163	-1.06537647	-2.2085	2.072976142	0.01670565
N20407	-1.06417356	-2.21	2.076728918	0.01670565
AA496283	-1.06243138	-2.253	2.120607539	0.01670565
AA169469	-1.06209971	-2.1735	2.046418029	0.01670565
AA858020	-1.05564497	-2.2895	2.168816286	0.01670565
AA490172	-1.05553732	-2.395	2.268986558	0.01670565
AA287190	-1.05367867	-2.1295	2.021014617	0.01670565
AA457544	-1.04745566	-2.0885	1.993879155	0.01670565
R76553	-1.04640695	-2.158	2.062295162	0.01670565
R56916	-1.04640068	-2.1015	2.008312911	0.01670565
AA131460	-1.04575705	-2.083	1.991858429	0.01670565
AA172050	-1.04496417	-2.187	2.092894726	0.01670565
AA609421	-1.04230704	-2.1005	2.015241115	0.01670565
R76614	-1.04080151	-2.12	2.03689175	0.01670565
AA670200	-1.04061244	-2.259	2.170837012	0.01670565
AA496009	-1.04011318	-2.121	2.039201151	0.01670565
AA418750	-1.03992459	-2.0975	2.016973165	0.01670565
N62244	-1.03963839	-2.039	1.961258864	0.01670565
R11047	-1.03783407	-2.081	2.005137485	0.01670565

AA45506	-1.03455339	-2.035	1.967032367	0.01670565
T89391	-1.03368713	-2.0885	2.020437267	0.01670565
T95274	-1.03296557	-2.0775	2.011199663	0.01670565
AA28466	-1.03253347	-2.251	2.180074616	0.01670565
AA16471	-1.03180811	-2.113	2.047861405	0.01670565
R78530	-1.03119267	-2.104	2.040355851	0.01670565
AA49695	-1.03083566	-2.0875	2.025056069	0.01670565
H73724	-1.02906208	-2.105	2.045552004	0.01670565
R10378	-1.02893419	-2.2185	2.15611458	0.01670565
N22980	-1.02616969	-2.0585	2.00600351	0.01670565
N62638	-1.0253414	-2.008	1.958372113	0.01670565
N52517	-1.02497722	-2.2005	2.146876976	0.01670565
W70343	-1.02410753	-2.167	2.115988737	0.01670565
AA44774	-1.02062394	-2.0515	2.010044962	0.01670565
H63077	-1.01599655	-2.0815	2.04872743	0.01670565
AA15674	-1.01155024	-1.988	1.965300316	0.01670565
AA44973	-1.01038406	-1.9965	1.975981296	0.01670565
AA17178	-1.01021807	-2.0335	2.012931714	0.01670565
N32295	-1.00999129	-2.038	2.017839191	0.01670565
R83836	-1.00466354	-2.0345	2.025056069	0.01670565
AA43226	-1.00464321	-2.0565	2.046995379	0.01670565
AA68357	-1.00461166	-2.2635	2.253109426	0.01670565
R98628	-1.00425178	-1.9925	1.9840642	0.01670565
N24076	-1.00274295	-2.2645	2.258305578	0.01670565
T95151	-1.00143487	-2.019	2.01610714	0.01670565
AA70422	-0.99785009	-1.987	1.991281078	0.01670565
R38539	-0.9937609	-2.1025	2.115700061	0.01670565
H73479	-0.99291681	-2.2125	2.228283364	0.01670565
N77263	-0.99125417	-2.062	2.08019302	0.01670565
AA48719	-0.9908803	-1.992	2.010333637	0.01670565
AA64465	-0.98740732	-1.9625	1.987528302	0.01670565
H68922	-0.98686117	-2.299	2.329608336	0.01670565
AA48756	-0.98573386	-2.0195	2.04872743	0.01670565
N74623	-0.98358081	-2.1775	2.213849607	0.01670565
H99676	-0.98156555	-2.1005	2.139948773	0.01670565
AA42099	-0.98144229	-2.1535	2.194219698	0.01670565
AA49598	-0.9806315	-2.1755	2.218468409	0.01670565
AA49704	-0.97234494	-1.98	2.036314399	0.01670565
AA39835	-0.97145146	-2.0295	2.089141949	0.01670565
AA85396	-0.97039592	-2.0385	2.100688954	0.01670565
H13424	-0.96583395	-1.9765	2.046418029	0.01670565
N26665	-0.96423703	-1.9415	2.013509064	0.01670565
N21338	-0.96251654	-2.4115	2.505411493	0.01670565
N25883	-0.96230127	-2.104	2.186425469	0.01670565
W69995	-0.96104007	-2.382	2.478564706	0.01670565
AA70144	-0.96020458	-1.9295	2.009467612	0.01670565
N64734	-0.95806615	-1.9335	2.018127866	0.01670565
H97146	-0.95597015	-1.945	2.034582349	0.01670565
AA41059	-0.95023676	-2.0565	2.164197484	0.01670565
AA43645	-0.94865644	-2.0005	2.108771858	0.01670565
N26108	-0.94481923	-2.0055	2.122628265	0.01670565
AA48544	-0.94376785	-2.0425	2.164197484	0.01670565
N94616	-0.94327539	-1.9995	2.119741513	0.01670565
AA41960	-0.94270098	-1.961	2.08019302	0.01670565
AA45637	-0.94073599	-1.9045	2.024478719	0.01670565
AA05853	-0.94000361	-2.446	2.602117663	0.01670565
T96083	-0.93893758	-1.9735	2.101843655	0.01670565
AA43036	-0.93743028	-1.943	2.072687466	0.01670565
AA41800	-0.93659406	-1.9145	2.044108628	0.01670565
AA03222	-0.93336071	-2.1	2.249933999	0.01670565
T74567	-0.9296992	-2.2595	2.430355958	0.01670565
W80701	-0.92755143	-1.99	2.1454336	0.01670565
AA45881	-0.92744165	-2.0575	2.218468409	0.01670565
R94808	-0.92665305	-1.9755	2.131865869	0.01670565
N98485	-0.92301257	-2.057	2.228572039	0.01670565
R49597	-0.92261812	-1.9115	2.071821441	0.01670565
N64741	-0.92251671	-2.2005	2.385322637	0.01670565
H27864	-0.92046607	-2.198	2.387920713	0.01670565
AA15141	-0.91660601	-2.7699	3.021909044	0.01670565
T64625	-0.91509	-1.949	2.129845143	0.01670565
R40481	-0.90974942	-1.883	2.069800715	0.01670565
AA40635	-0.90870905	-1.8575	2.044108628	0.01670565
H94469	-0.90859316	-1.889	2.079038319	0.01670565
R66310	-0.90769311	-1.917	2.111947285	0.01670565
AA70480	-0.90598493	-1.9215	2.120896214	0.01670565
R26756	-0.9039483	-1.895	2.096358827	0.01670565
N42770	-0.9020393	-1.866	2.068646015	0.01670565
W73874	-0.90019987	-1.9295	2.143412874	0.01670565

R33402	-0.89556599	-2.395	2.674286447	0.01670565
R52965	-0.89157257	-1.902	2.133309245	0.01670565
AA45214[illegible]	-0.88989839	-1.8545	2.083945797	0.01670565
AA42766[illegible]	-0.88850909	-1.9265	2.168238936	0.01670565
AA49004[illegible]	-0.88140984	-1.862	2.112524635	0.01670565
H94043	-0.88086348	-1.87	2.12291694	0.01670565
AA45750[illegible]	-0.88081652	-1.9205	2.180363292	0.01670565
N63635	-0.87841081	-1.844	2.099245579	0.01670565
AA59865[illegible]	-0.87569321	-1.8795	2.146299626	0.01670565
N33322	-0.8724874	-1.8565	2.127824417	0.01670565
W73810	-0.87140741	-1.8215	2.09029665	0.01670565
H77697	-0.86969306	-1.897	2.181229317	0.01670565
AA44936[illegible]	-0.86391663	-2.7858	3.224616723	0.01670565
R38459	-0.86237427	-1.8305	2.122628265	0.01670565
N66120	-0.86124793	-1.983	2.302472874	0.01670565
AA62040[illegible]	-0.85956597	-1.863	2.167372911	0.01670565
R28660	-0.85859808	-1.8785	2.187868845	0.01670565
N66144	-0.85750082	-1.7855	2.082213746	0.01670565
W92703	-0.85641721	-2.1615	2.523886702	0.01670565

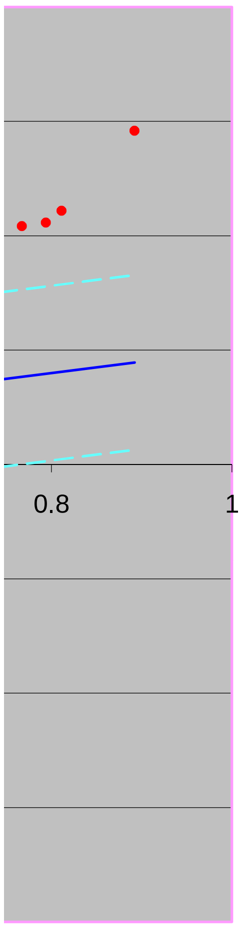

Supplement: Additional file 3 — Table S1-8. SAM Analysis. [file 1471-2164-10-326-S3.pdf]
